# The Effect of Dietary Interventions on Chronic Inflammatory Diseases in Relation to the Microbiome: A Systematic Review

**DOI:** 10.3390/nu13093208

**Published:** 2021-09-15

**Authors:** Carlijn A. Wagenaar, Marieke van de Put, Michelle Bisschops, Wendy Walrabenstein, Catharina S. de Jonge, Hilde Herrema, Dirkjan van Schaardenburg

**Affiliations:** 1 Amsterdam Rheumatology and Immunology Center, Reade, 1056 AB Amsterdam, The Netherlands; m.vd.put@reade.nl (M.v.d.P.); m.bisschops@reade.nl (M.B.); w.walrabenstein@reade.nl (W.W.); d.v.schaardenburg@reade.nl (D.v.S.); 2 Amsterdam UMC, Amsterdam Medical Center, 1105 AZ Amsterdam, The Netherlands; 3 Department of Radiology and Nuclear Medicine, Amsterdam Gastroenterology Endocrinology Metabolism, Amsterdam UMC, University of Amsterdam, 1105 AZ Amsterdam, The Netherlands; c.s.dejonge@amsterdamumc.nl; 4 Department of Experimental Vascular Medicine, Amsterdam University Medical Centers (UMC), Academic Medical Center, 1105 AZ Amsterdam, The Netherlands; h.j.herrema@amsterdamumc.nl

**Keywords:** chronic inflammation, inflammatory disease, microbiome, diet, fiber

## Abstract

Chronic inflammation plays a central role in the pathophysiology of various non-communicable diseases. Dietary interventions can reduce inflammation, in part due to their effect on the gut microbiome. This systematic review aims to determine the effect of dietary interventions, specifically fiber intake, on chronic inflammatory diseases and the microbiome. It aims to form hypotheses on the potential mediating effects of the microbiome on disease outcomes after dietary changes. Included were clinical trials which performed a dietary intervention with a whole diet change or fiber supplement (>5 g/day) and investigated the gut microbiome in patients diagnosed with chronic inflammatory diseases such as cardiovascular disease (CVD), type 2 diabetes (T2DM), and autoimmune diseases (e.g., rheumatoid arthritis (RA), inflammatory bowel disease (IBD)). The 30 articles which met the inclusion criteria had an overall moderate to high risk of bias and were too heterogeneous to perform a meta-analysis. Dietary interventions were stratified based on fiber intake: low fiber, high fiber, and supplemental fiber. Overall, but most pronounced in patients with T2DM, high-fiber plant-based dietary interventions were consistently more effective at reducing disease-specific outcomes and pathogenic bacteria, as well as increasing microbiome alpha diversity and short-chain fatty acid (SCFA)-producing bacteria, compared to other diets and fiber supplements.

## 1. Introduction

Chronic inflammatory diseases, defined as non-infectious diseases where chronic inflammation is a key component of the etiology and progression of disease, are a main cause of morbidity worldwide reducing quality of life and longevity [1]. Inflammation plays a large role in metabolic syndrome, type 2 diabetes (T2DM), and cardiovascular disease (CVD) [1]. It is also fundamental to autoimmune diseases such as rheumatoid arthritis (RA), which are associated with increased risk of metabolic syndrome, T2DM, and CVD [2,3,4]. C-reactive protein (CRP) is an inflammatory marker and elevated levels are related to an increased risk of chronic disease and all-cause mortality [5,6]. Consequently, reducing inflammation leads to a reduction of cardiovascular events and its risk factors [7,8].

Lifestyle factors, such as smoking, unhealthy diet, and physical inactivity are key risk factors for chronic inflammation and modifications hereof could potentially prevent 70–90% of various chronic diseases [1,9]. Of these risk factors, dietary behavior plays the largest role in deaths and disability-adjusted life years [10]. Mediterranean diets, diets high in fruits, vegetables, and other plant foods, as well as high-fiber diets (including fiber supplementation) are associated with reduced levels of inflammation [11,12].

One of the mechanisms involved in the pro- or anti-inflammatory effects of diet is the intermediate impact of diet on the composition and metabolic activity of the gut microbiome [13]. The gut microbiome consists of multiple microorganisms such as bacteria, viruses, protozoa, and fungi present in the large intestine [14]. The microbiome also has various functions including digestion, metabolite synthesis, and communication with the immune system to aid its development and modulate inflammatory responses [13,15]. The composition and ratio of bacterial species can differ significantly based on many factors, in particular health status, whereby a microbiome with a greater diversity of microorganisms has been shown to be associated with health [14,16].

Furthermore, gut microbiome dysbiosis, defined as an imbalance in the amount and function of the gut microbial community, is correlated with chronic inflammatory diseases such as autoimmune and metabolic diseases [16,17]. Growing evidence suggests microbiome dysbiosis can disrupt intestinal barrier integrity, including the mucus layer and epithelial cell junctions, resulting in increased intestinal permeability [13,18]. This consequently allows for the translocation of harmful microbiome-derived and environmental components into the mucosal layer and the systemic circulation thus fueling host immune responses and chronic inflammatory disorders [13,18].

**Figure 1:**
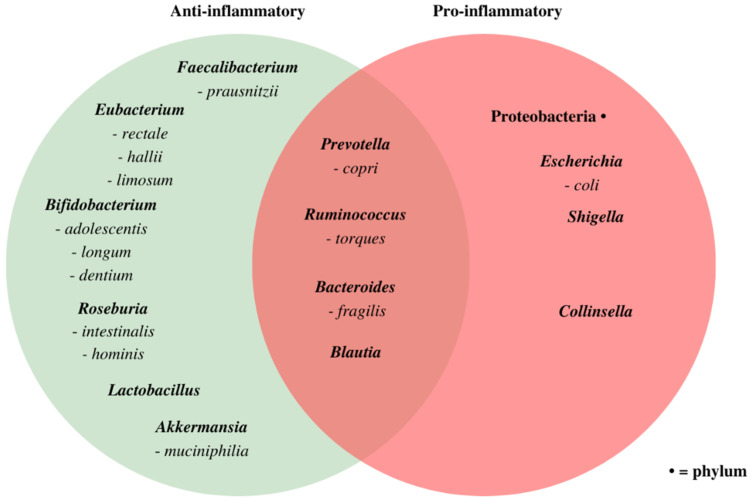
Venn diagram grouping various bacteria genus (bold) and species as anti-inflammatory (left circle), pro-inflammatory (right circle), or both (overlap between circles) depending on their abundance and environment [19,20,21,22,23,24,25,26,27,28,29,30,31,32,33,34,35,36,37]. Included microbiota were selected based on the frequency of appearance in the included articles of this systematic review to allow for comparisons between articles.

While a richer and more diverse microbiome is characterized as healthy, the optimal composition of a healthy gut microbiome is still unclear and seems to vary per individual [16,17]. Summarized in Figure 1 are specific bacteria genera and species usually associated with health, in part due to their anti-inflammatory effects, and on the contrary, those known as opportunistic pathogens able to induce pro-inflammatory responses [19,20,21,22,23,24,25,26,27,28,29,30,31,32,33,34,35,36,37]. An overlapping characteristic of the anti-inflammatory beneficial bacteria is their ability to produce short-chain fatty acids (SCFA), specifically butyrate, acetate, and propionate [38]. In vitro and mouse models have shown SCFAs modulate inflammation in the gut by improving transepithelial resistance, modification of various signaling pathways, and inhibiting pro-inflammatory cytokines while up-regulating anti-inflammatory cytokines [20]. On the other hand, Proteobacteria, such as *E. coli* and *Shigella*, and *Collinsella* are associated with chronic inflammatory diseases [27,28]. For certain bacteria, shown in the overlapping area of the Venn diagram of Figure 1, whether their functions are beneficial or harmful to the host depends on factors such as their abundance and their environment [29].

Diet is one of the most powerful ways to alter the gut microbiome [17]. High-fiber diets (e.g., vegetarian, vegan, Mediterranean), which are low in red meat and higher in unsaturated fatty acids, are associated with a more beneficial microbiome composition, an increased microbial diversity, and more health-promoting bacteria (e.g., *Bifidobacteria*, *Lactobacillus*, *Prevotella*, *Eubacterium*, *Roseburia*) as well as higher levels of SCFAs [39,40,41,42]. Contrarily, Western diets, characterized by high animal fat and protein and low fiber, show an overall decrease in total bacteria, *Bifidobacteria*, *Lactobacillus*, and *Eubacterium*, while increasing pathogenic Proteobacteria [41]. Daily fiber intake plays a vital role in the microbiome changes associated with diet [43]. In the gut, non-digestible carbohydrates are fermented by the microbiome to form SCFAs, and specific types of dietary fiber, known as prebiotics, are capable of selectively stimulating the growth of bacteria in the colon and benefitting host health [41,43,44].

Dietary interventions aimed at reducing inflammatory chronic diseases and improving the microbiome are promising. Yet, the field is still evolving and due to a large heterogeneity of studies, drawing concrete conclusions has proven difficult in the past [45]. Consequently, this systematic review aims to determine the effect of dietary interventions on chronic inflammatory diseases and the microbiome and the extent to which the microbiome plays a role in the relationship between dietary interventions and chronic inflammatory diseases.

## 2. Materials and Methods

### 2.1. Search Strategy

This systematic review was written following the Preferred Reporting Items for Systematic Reviews and Meta-Analyses (PRISMA) guidelines [46]. The review was registered prior to its start in the PROSPERO international prospective register of systematic reviews (CRD42021229471). A systematic search was performed in the electronic databases: PubMed, Cochrane, Embase and CINAHL up to July 2021, no date restrictions were used. Search terms related to diet (e.g., “Diet,” “Nutrition,” “Fiber”), microbiome (e.g., “Microbiota,” “Gastrointestinal microbiome”) and chronic inflammatory diseases (e.g., “Autoimmune disease,” “Inflammation,” “Diabetes,” “Heart disease”) were combined. See Supplement S1 for the complete search strategy per database. Only English language articles were included, and duplicate articles were excluded.

### 2.2. Study Selection

**Figure 2:**
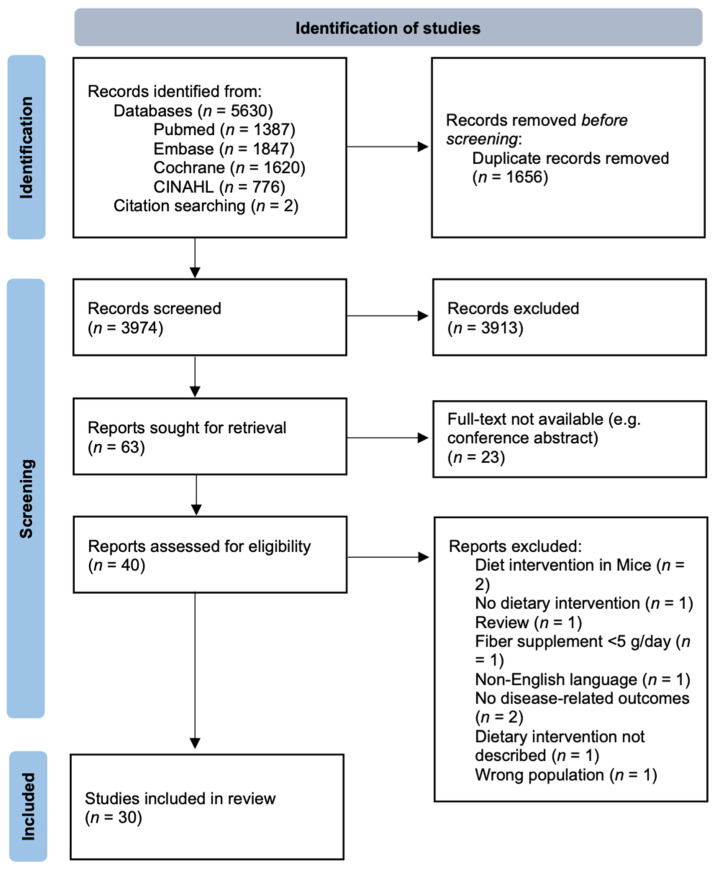
Flow diagram of the systematic literature search for dietary intervention, chronic inflammatory disease, and microbiome based on the PRISMA method [46].

Three authors (C.A.W., M.v.d.P., and M.B.) used the eligibility criteria described below to screen studies for inclusion based on title and abstract. Rayyan (https://rayyan.qcri.org/, accessed on 7 July 2021) was used to record decisions. Reviewers performed the screening independently and were blinded to each other’s decisions. Disagreements were resolved via discussion and consensus. Studies that passed the initial screening were further screened based on their full text. Studies which fulfilled the inclusion criteria then underwent data extraction (Figure 2).

### 2.3. Eligibility Criteria

Randomized clinical trials (RCTs) and clinical trials with both disease-specific and gut microbiome outcomes were included. Only studies with whole diet interventions, including fiber supplements (≥5 g/day) or synbiotics (prebiotic (≥5 g/day) and probiotic combined) were included. It was decided to include trials with a fiber supplementation of ≥5 g/day, since such an increase (+25% of mean intake) of one macro nutrient changes the whole diet substantially [47]. Studies conducted in populations ≥18 years old with cardiovascular disease (CVD), diabetes mellitus (DM), and autoimmune diseases (e.g., rheumatoid arthritis (RA), inflammatory bowel diseases (IBD)) were included. These disease groups were chosen from the larger array of chronic diseases included in the search strategy (Supplement S1) as they have the strongest empirical evidence for the role of chronic inflammation in their disease onset or progression [1]. Although metabolic syndrome has a strong association with chronic inflammation, it was not included as a disease in this review as it is a collection of risk factors for other diseases such as T2DM and CVD. Healthy participants and other health conditions were excluded.

### 2.4. Data Extraction

Data extraction was performed by one author (C.A.W., M.v.d.P., or M.B.) and checked by a second. Disagreements were resolved via discussion and consensus. Microsoft Excel was used for data extraction and management. The data extracted from the studies included: author, year, country, study design, population characteristics (age, gender, disease being studied), sample size (total and per group), dietary intervention(s) and control group diet, methods for data collection, dietary assessment method and adherence to dietary intervention, study duration and follow-up, disease-specific outcomes, and gut microbiome outcomes. Given the heterogeneity between studies and their reported outcomes, a selection of outcomes was made per disease type as well as for the microbiome based on the most frequently recurring and most clinically relevant outcomes to allow for comparison between studies. The following disease-specific outcomes were selected (main disease-specific outcome in italics): *clinical activity index* (e.g., Crohn’s Disease activity index, Partial Mayo Score) and fecal calprotectin (FC) for IBD, *fasting blood glucose* (FBG) and HbA1C for T2DM, *disease activity index* (e.g., Disease Activity Score for 28 Joints (DAS28) or RA disease improvement index) for RA, and *Low density lipoproteins* (LDL), systolic blood pressure (SBP), high density lipoproteins (HDL), total cholesterol (TC), triglycerides (TG) for CVD. When available, CRP was selected to assess inflammatory levels for all disease populations. With respect to the gut microbiome outcomes, alpha diversity (diversity of bacteria in one sample), SCFAs, and specific bacteria were extracted. Classification of the microbiome using Phyla, class, order, or family is non-specific as within these classifications different genera and species can have varying effects on the host. As a result, bacterial outcomes at genus and species level were preferred.

### 2.5. Risk of Bias Assessment

For the included articles, risk of bias was assessed by one author (C.A.W., M.v.d.P., or M.B.) and checked by a second using the Revised Cochrane Risk-of-Bias tool for Randomized Trials (RoB 2) with additional considerations for crossover trials, and the ROBINS-I assessment tool (the Risk of Bias In Non-randomized Studies of Interventions) [48,49]. Assessments were performed based on the disease-specific outcome.

### 2.6. Data Presentation

To create an overview and compare the study outcomes, (percentage) relative changes of outcomes were calculated between intervention and control groups at the end of the trial or within the intervention group at baseline and end of the trial (for studies with no control group, or a control group with a non-relevant comparator intervention (e.g., fasting)). Percentage relative changes were calculated as the percentage difference of the numerical outcome of the intervention group when compared with the outcome of the control group at the end of the intervention, relative to the control group. For studies without a control group the percentage difference between the outcome and baseline value was calculated, relative to the baseline value. When combining these results from the different disease categories (CVD, T2DM, RA, and IBD), the main disease-specific outcome was used when available. To accommodate for different amounts of participants in the trials, percentage relative changes are presented as weighted averages. Dietary interventions were grouped into three categories to allow for comparisons between studies: low fiber (e.g., specific carbohydrate diet, FODMAP diet, and low-carb diet), high fiber (e.g., Mediterranean, vegetarian, vegan, high-fiber, macrobiotic), and supplemental fiber (e.g., inulin, galacto-ogliosaccharide (GOS), fructo-ogliosaccharides (FOS), psyllium, synbiotic, high-fiber functional foods). Low-fiber dietary interventions were defined as studies with interventions that deliberately reduced (fermentable) fiber or (non-soluble) carbohydrate intake: a low FODMAP dietary pattern restricts dietary intake of short chain carbohydrates (Fermentable Oligo-, Di-, Mono-saccharides, And Polyols), the specific carbohydrate diet restricts the intake of disaccharides and polysaccharides, and a low residue diet limits high-fiber foods which are indigestible and thus contribute to fecal bulk (e.g., whole grains, nuts, seeds, raw or dried fruits and vegetables) [50,51]. On the other hand, studies with high-fiber dietary interventions deliberately increased fiber intake, while studies which gave fiber supplements were assigned to the “supplemental fiber” group. Dividing diet types based on daily fiber intake was not possible as not every study quantitatively reported fiber intake.

**Figure 3:**
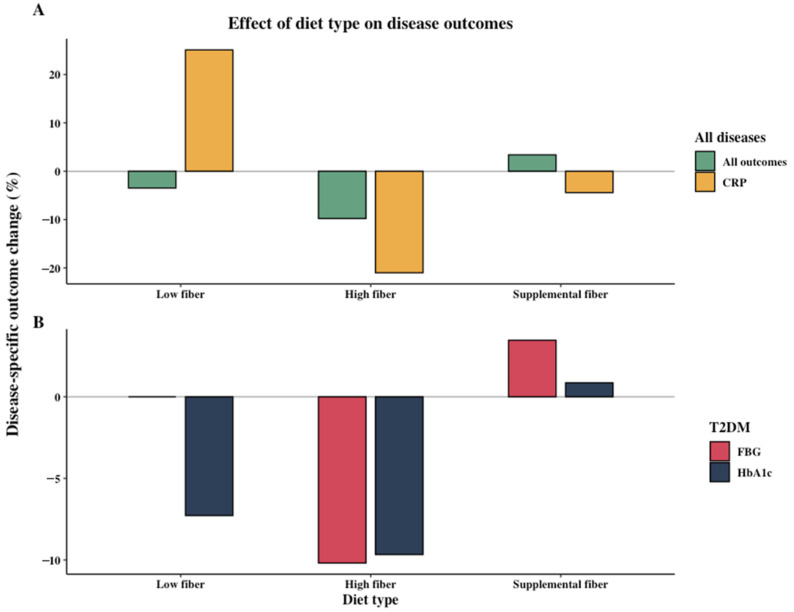
Effect of diet type (low fiber, high fiber, and supplemental fiber) on disease-specific outcomes for (**A**) all diseases combined and (**B**) Type 2 diabetes (T2DM). Percentage relative change between intervention group and control group or end intervention and baseline is shown. Averages are weighted based on number of participants in the intervention group. Statistical significance of outcomes not incorporated. (**A**) All disease outcomes (low fiber: 2 studies, *n* = 46; high fiber: 12 studies, *n* = 180; supplemental fiber: 11 studies, *n* = 246); C-reactive protein (CRP) (low fiber: 1 study, *n* = 24; high fiber: 7 studies, *n* = 105; supplemental fiber: 5 studies, *n* = 136). (**B**) Fasting blood glucose (FBG) (low fiber: 0 studies, *n* = 0; high fiber: 4 studies, *n* = 72; supplemental fiber: 6 studies, *n* = 150); Hemoglobin A1c (HbA1c) (low fiber: 1 study, *n* = 25; high fiber: 4 studies, *n* = 72; supplemental fiber: 6 studies, *n* = 163).

**Figure 4:**
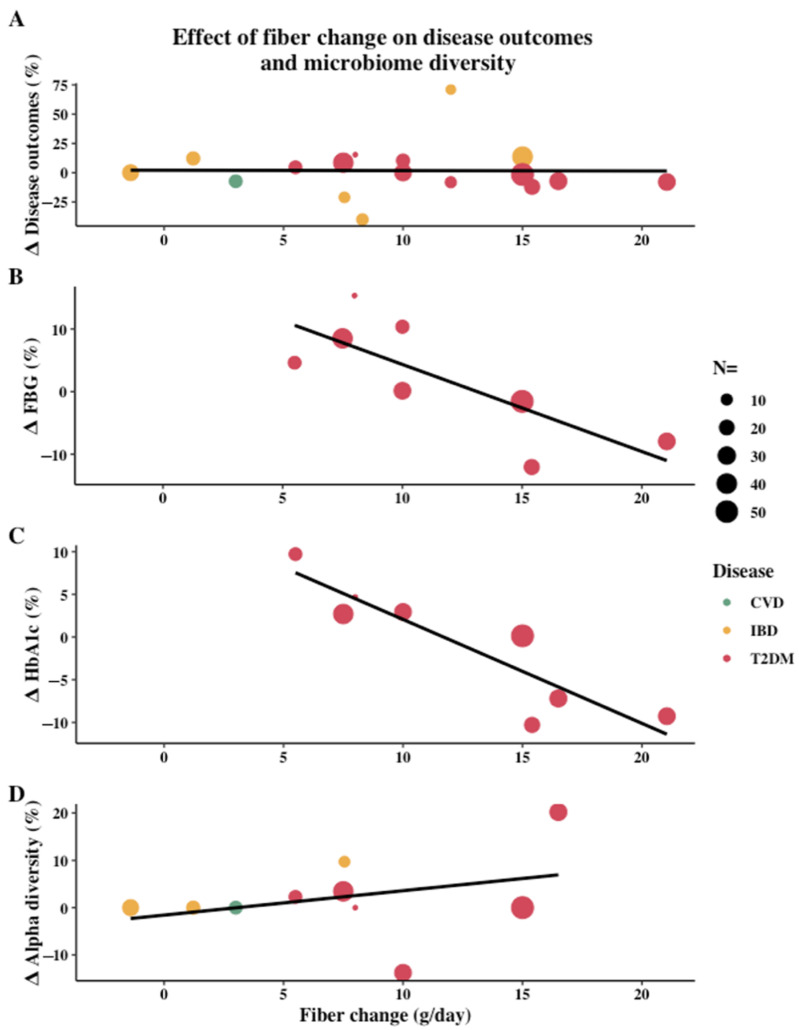
Effect of change in daily fiber intake (g/day) on (**A**) all diseases (type 2 diabetes (T2DM) (red points), inflammatory bowel disease (IBD) (yellow points), and cardiovascular disease (CVD) (green points)) with their respective disease-specific outcomes, (**B**) fasting blood glucose (FBG) in T2DM patients, (**C**) hemoglobin A1c (HbA1c) in T2DM patients, and (**D**) microbiome alpha diversity in all diseases. Percentage relative change between intervention group and control group or end intervention and baseline is used to determine change in disease and microbiome outcomes, as well as fiber change. Only studies which reported the amount of fiber intake were included. Size of the points are based on the number of participants (*n* =) in the intervention group. Statistical significance of outcomes not incorporated. In (**D**), changes reported as “non-significant” without numerical results were plotted as a change of 0.00%.

**Figure 5:**
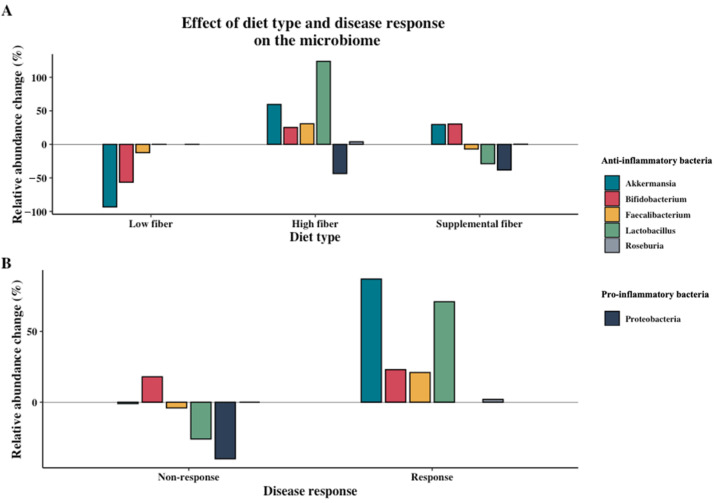
(**A**) Effect of diet type (low fiber, high fiber, and supplemental fiber) and (**B**) disease response on changes of bacteria in the gut microbiome. Percentage relative change between intervention group and control group or end intervention and baseline is shown. Averages are weighted based on number of participants in the intervention group (data not shown). Statistical significance of outcomes not incorporated.

The groups of diet types and percentage relative changes were compared in various ways and presented in Figures 3–5 to provide an overview of the data and answer various sub-questions. These questions included whether there was a general trend between diet type and disease-specific outcomes (Figure 3), whether there was a trend between the amount of daily fiber intake and disease-specific or microbiome outcomes (Figure 4), and if there was a trend between the effect of different diet types on specific changes in the microbiome (Figure 5A).

To explore the association between disease response and microbiome changes due to dietary intervention, the studies were categorized as “non-response,” defined as no statistically significant improvement or a negative change in the disease-specific outcome(s), or “response,” defined as a significant improvement in the disease-specific outcome(s). To form a hypothesis of whether microbiome changes could be a mediating factor between dietary interventions and changes in disease outcomes, percentage relative changes of bacteria in the gut microbiome were graphed with “response” or “non-response” studies (Figure 5B).

No further statistical analysis was performed using the results. Due to the low number of selected studies and the diversity of outcome measures, it was not possible to perform a statistical meta-analysis.

## 3. Results

The search strategy resulted in 5630 articles from PubMed (*n* = 1387), Embase (*n* = 1847), Wiley/Cochrane library (*n* = 1620), and CINAHL (*n* = 776). Two articles were additionally identified through references. In total, 1656 duplicate articles were removed, and 3913 articles were excluded after double-blind screening of title and abstract. Overall, 63 articles were sought for the full-text screening, of which 40 were available as a full-text article and consequently 30 articles (covering 29 studies) were found to comply with the eligibility criteria of this review (full text not available for 23 articles). See Figure 2 for an overview of the search and selection process. Risk of bias was moderate (38% of studies) to high (48% of studies) in most studies mainly due to uncontrolled confounders in the non-randomized studies, no trial register, or missing outcomes due to exacerbated disease which potentially impacted the study outcome (Supplementary Figures S1–S3). Table 1 shows a summary of all included articles grouped by disease type.

### 3.1. Effect of Dietary Interventions on Disease-Specific Outcomes and the Microbiome

#### 3.1.1. Low-Fiber Dietary Interventions

Three articles studied the effect of a lower fiber dietary intervention, including low FODMAP and specific carbohydrate diets, on disease activity and microbiome composition in patients with IBD [52,53,54]. These studies did not show a significant clinical difference between the intervention groups and the control groups at the end of the intervention period. However, Cox et al. and Halmos et al. did show significant harmful alterations of the microbiome possibly due to the decrease in fiber intake of 1.4 and 2.5 g/day, respectively. Specifically, Cox et al. showed a significant decrease in SCFA and relative abundances of *Bifidobacterium* spp. and *F. prausnitzii*, while Halmos et al. measured a statistically significant decrease in relative abundances of *A. muciniphilia* and butyrate-producing *Clostridium coccoides* group in the low FODMAP group compared to the control group [52,53].

In contrast, Ren et al. showed a beneficial clinical effect on glucometabolic parameters after a three-month RCT in T2DM patients using a low-carbohydrate diet [55]. In this study, both intervention and control group received the same initial diet, but 150 g/day of carbohydrate foods were replaced with 56 g/day of almonds in the low-carbohydrate group. Although both groups showed significant improvements in HbA1c after three months, the low-carbohydrate group had a significantly lower score at the end of the intervention period (6.88 ± 0.12% vs. 7.42 ± 0.12%, *p* < 0.01). Additionally, the low-carbohydrate group had a higher relative abundance of *Roseburia*, *Ruminococcus*, and *Lactobacillus*, and less *Eubacterium* compared to the control diet.

#### 3.1.2. High-Fiber Dietary Interventions

##### Mediterranean Diet

Six studies investigated the effects of a Mediterranean-inspired diet whereby overall there was a trend towards improvements in disease-specific outcomes and microbiome composition. In IBD patients, Marlow et al. showed a non-significant reduction of CRP after six weeks and a shift towards a more beneficial microbiota by increasing the relative abundances of butyrate-producing *Clostridium coccoides* (+2.01%) and *Clostridium leptum* (+2.66%) groups and decreasing Proteobacteria (−0.45%) [56]. In the study by Zhang et al., on the other hand, there was no clinical improvement in IBD patients, and, despite the dietary intervention, participants only increased their daily fiber intake marginally (+1.22 g/day, *p* = 0.68) [58]. Yet at the end of the intervention, the microbiome was modified beneficially via an increase in the mean relative abundance of *Faecalibacterium* (3.35 ± 5.4% vs. 5.58 ± 7.4%, *p* = 0.04), *Bifidobacterium* (1.09 ± 2.2% vs. 1.47 ± 2.4%. *p* = 0.06), and a trend towards a decrease in Proteobacteria (7.23 ± 1.2% vs. 2.69 ± 3.4%, *p* = 0.51).

A German research group also performed two clinical trials in RA patients. For both studies, a two-week normocaloric, mostly vegetarian (1–2 servings of meat/week), whole-grain Mediterranean diet was prescribed along with a multidisciplinary integrative lifestyle treatment including exercise, physical therapy, and stress management. In the 2005 study by Michalsen et al., there was a non-significant reduction in DAS28 (−0.2 DAS28 points, *p* = 0.425) and trend towards an increase in *Lactobacillus* and *Bifidobacterium* in seven patients [64]. Later, in a larger population (*n* = 28), Abendroth et al. (2010) showed a decrease in DAS28 score (*p* < 0.001), CRP, and SCFA (22.9 ± 13.8 to 20.4 ± 9.8 μmol/g) [65].

Furthermore, in a population with T2DM patients, Liu et al. performed a six-month intervention with a low-fat Mediterranean diet that significantly reduced FBG and HbA1c [71]. Although both *Roseburia* and *Lachnospira* increased at one month, only *Pseudomonas* increased significantly after six months. In a 12-week uncontrolled pilot study, Ismael et al. also studied a Mediterranean-based diet in T2DM patients and showed a decrease in both FBG (131.63 ± 8.53 vs. 122.50 ± 9.42 mg/dL, *p* = 0.581) and HbA1c (7.53 ± 1.07 vs. 6.86 ± 0.85%, *p* = 0.025) [72]. There was also a trend towards an increase in alpha diversity (*p* = 0.47) and relative abundances of *Bacteroides*, *Ruminococcus*, *Akkermansia*, and *Roseburia*, while the relative abundance of *Faecalibacterium* and *Prevotella* decreased.

##### High-Fiber Diet

Overall, studies with a high-fiber dietary intervention resulted in beneficial improvements of clinical and microbiome outcomes. First, Fritsch et al. studied the effects of a low-fat, high-fiber diet compared to an improved Standard American Diet (iSAD) in ulcerative colitis (UC) patients (+7.55 g/day of fiber in high-fiber group) [57]. Both groups had a significant decrease in Partial Mayo score while the high-fiber group also had a significant reduction of CRP. When comparing the groups after the intervention the high-fiber group had a lower Partial Mayo score (*p* = 0.63), fecal calprotectin (*p* = 0.19), and CRP (*p* = 0.21) and a higher relative abundance of *F. prausnitzii* (*p* = 0.04) and *Prevotella* (*p* = 0.08). In another study, Zhao et al. explored the effects of a high-fiber diet, consisting of meals with fresh vegetables, fruits, nuts, and formulas containing whole grains, traditional Chinese medicinal foods, and prebiotics [70]. Overall, the high-fiber group consumed 21.04 more g/day of fiber than the control group which received standard dietary and exercise advice for T2DM. After 12 weeks, both the high-fiber and control groups had a significantly lower FBG and HbA1c compared to baseline. Between group analysis resulted in a non-significant lower FBG and a significant lower HbA1c in the intervention group. The high-fiber group had a greater beta-diversity (*p* < 0.001) and a larger abundance of *F. prausnitzii* and *Eubacterium rectale*, yet had less *Lactobacillus reuteri* and *Bifidobacterium longum*.

Lastly, in a sub-group of a larger RCT comparing a low-fat, high complex carbohydrate diet with a Mediterranean diet in CVD patients, Haro et al. (2016) showed that the high complex carbohydrate group had a non-significantly lower total cholesterol, LDL, HDL, and an increase in triglycerides after one year [66]. Compared to the Mediterranean diet group, the high complex carbohydrate diet resulted in a greater abundance of *Prevotella* (*p* = 0.028) and *F. prausnitzii* (*p* = 0.516). In a different sub-group of the same larger RCT, Haro et al. (2017) showed both diets were effective at lowering triglycerides, and when grouped together, both diets resulted in beneficial changes of microbiome composition [67].

##### Vegetarian and Vegan Diet

Vegetarian, vegan, and macrobiotic dietary interventions all resulted in significant improvements in disease-specific outcomes and trends towards a more beneficial microbiome composition. In RA patients, a 13-month intervention by Peltonen et al. (1994) compared a vegan (first 3.5 months) and later lacto-vegetarian diet with an ordinary omnivore diet [62]. After one month, patients within the intervention group showed significant clinical improvements. Compared to the control group, after 13 months the intervention group had significantly less tender and swollen joints, pain, erythrocyte sedimentation rate (ESR), and CRP. A second study in RA patients by Peltonen et al. (1997) compared a raw vegan diet to an ordinary omnivorous diet [63]. After four weeks, the raw vegan group had a significant increase in the Disease Improvement Index (including changes in pain, health assessment questionnaire, number of swollen joints, number of tender joints, subjective evaluation of improvement, and ESR) compared to the omnivorous group. In both studies, changes in the gut microbiome were evaluated using direct gas-liquid chromatography (GLC) to measure bacterial cellular fatty acid (CFA) profiles of stool samples. In the 1994 study, a statistically significant difference in CFA profiles was found when comparing baseline samples to the samples taken throughout the intervention period (*p* < 0.0005), as well as a significant difference in similarity index (calculated by comparing fatty acid spectra between individual samples) between the lacto-vegetarian and vegan diet. Additionally, at the end of the 1997 intervention, the raw vegan group had a lower similarity index to baseline samples than the control group (*p* < 0.001). A decrease in similarity index could indicate an increase in beta-diversity between samples.

Candela et al. performed a three-week, in-patient RCT in patients with T2DM (*n* = 40) [69]. The intervention group received a macrobiotic diet consisting of whole grains, vegetables, and legumes with roasted green tea as its main beverage. The control group followed a Mediterranean-inspired diet based on recommendations for T2DM. For both diets, five meals were provided per day and calories were restricted to 1700 kcal/day and 1900 kcal/day for women and men, respectively. On average, at the end of the intervention the macrobiotic group consumed 15.4 more g/day of fiber than the control group. After three weeks there was a significant decrease in FBG (*p* = 0.002) and CRP (*p* = 0.03) between intervention and control groups, while the reduction in HbA1c was non-significant (*p* = 0.3). Compared to the control group, the macrobiotic diet resulted in a non-significant increase of alpha diversity and showed a greater increase of *Akkermansia*, while *Collinsella* decreased.

Lastly, in CVD patients, Djekic et al. compared a lacto-ovo vegetarian diet with a conventional diet based on the average meat consumption in Sweden (146 g/day) [68]. In this randomized cross-over study, participants consumed 3 g/day more fiber when following the vegetarian diet. Both diets led to a significant reduction of LDL, HDL, and total cholesterol, while when comparing the diets, the vegetarian diet group had a lower LDL (*p* = 0.02) and total cholesterol (*p* = 0.01). There was a trend towards a higher abundance of *Roseburia*, *Bifidobacterium*, *lactobacillus*, and a decrease in *Collinsella*, yet only *Akkermansia* was significantly increased after the vegetarian diet compared to the conventional diet.

#### 3.1.3. Supplemental Fiber Dietary Interventions

In total, 11 studies were performed with supplemental fiber interventions in both IBD and T2DM populations. Studies investigating <10 g/day of fiber supplementation did not show significant changes in disease-specific outcomes in T2DM patients [73,79,81]. Additionally, these interventions only resulted in changes in the microbiome when combined with a probiotic. Specifically, Kanazawa et al. found significantly more *Lactobacillus* and *Bifidobacterium* in the synbiotic group (containing *Lactobacillus parasei*, *Bifidobacterium breve*, and 7.5 g/day of GOS) compared to placebo, as well as a significant increase in *Akkermansia muciniphilia* compared to baseline [81]. Horvath et al. also explored the effects of a synbiotic (*Lactobacillus* and *Bifidobacterium* spp. with 8 g/day of GOS and FOS) in T2DM patients, whereby *Lactobacillus brevis* increased after supplementation [79].

On the other hand, in UC patients Kanauchi et al. showed a four-week open-label study comparing supplementation with 20–30 g/day of germinated barley foodstuff (6.66–10 g/day fiber) effectively lowered the Clinical Activity Index (Lichtiger method) (*p* < 0.045) and caused beneficial changes to the microbiome [59].

Four studies used fiber supplements with 10–15 g/day of fiber with varying effects. After supplementation with 10 g/day of GOS in T2DM patients, Gonai et al. reported no change in disease-specific outcomes [74]. Within the intervention group there was a significant decrease in alpha diversity, while Bifidobacteriaceae increased significantly. In another study, Roshanravan et al. supplemented T2DM patients with 10 g/day inulin [75,76]. After 45 days there was no significant change in FBG from baseline, yet hs-CRP significantly decreased compared to baseline and the placebo group. Additionally, compared to the placebo group there was a statistically significant increase in *A. muciniphilia*. Furthermore, after a four-week intervention with 12 g/day psyllium supplementation, Lee et al. showed that the fiber supplement did not induce statistically significant changes in FBG in T2DM patients, while there was a statistically significant reduction of *Blautia*, *Eubacterium*, *Bifidobacterium longum*, and *Enterobacter soli* [77]. Moreover, Furrie et al. compared a synbiotic supplement (*Bifidobacterium longum* with 12 g/day inulin-oligofructose) to placebo in UC patients [60]. Within the intervention group there was a reduction in the Clinical Activity Index (Walmsley method) and CRP at the end of the four-week study, although the placebo group had a lower Clinical Activity Index after four weeks (Synbiotic 5.3 (3.4) vs. Placebo 3.1 (2.5) points). When compared to placebo, the synbiotic increased the amount of *Bifidobacteria*.

Lastly, three studies evaluated the effects of supplementing ≥ 15 g/day fiber, of which only one showed beneficial effects on disease and microbiome outcomes. In CD patients, Benjamin et al. reported no significant difference in disease-specific or microbiome outcomes between intervention (15 g/day FOS) and control groups [61]. Comparatively, Reimer et al. showed that a 52-week intervention with 15–20 g/day of highly viscous polysaccharides led to no significant difference in the change of FBG or HbA1c between the intervention and control group [80]. At the end of the intervention, the intervention group had significantly less *F. prausnitzii* (*p* = 0.038) and *Lactobacillus* (*p* = 0.035) and more *Collinsella.* compared to the control group (*p* = 0.012). On the other hand, in T2DM patients Medina-vera et al. demonstrated that a functional food intervention consisting of 16.5 g/day of fiber (including dehydrated nopal, chia seeds, soy protein, and inulin) led to a larger percentage change from baseline of HbA1c (−7.20%, *p* < 0.05) and CRP (−13%, *p* < 0.01) (*p* <0.05) than placebo [78]. Additionally, at the end of the study, the functional food group had an increase in alpha diversity (*p* < 0.05), *F. prausnitzii* (+ 34%), *A. muciniphilia* (+ 125%), as well as a 13% decrease in *P. copri* compared to the control group.

### 3.2. Effect of Dietary Intervention Type and Fiber

To create an overview and view trends of the effects of different diets on disease-specific and microbiome outcomes, the included studies were stratified based on diet type (low fiber, high fiber, and supplemental fiber). For all disease populations combined, high-fiber diets were most effective at lowering disease outcomes as well as CRP compared to both low-fiber and supplemental fiber diets (Figure 3A). Specifically, in T2DM patients both FGB and HbA1c were reduced more after high-fiber dietary interventions (Figure 3B). Similarly, high-fiber diets lowered the disease-specific outcomes of IBD patients (−5.21%, 3 studies, *n* = 32), while the low-fiber and supplemental fiber studies did not change or rather increased disease activity (0.00% (1 study, *n* = 24) and +10.39% (3 studies, *n* = 58), respectively).

Furthermore, daily fiber intake varied substantially between studies. To determine if the amount of fiber intake was associated with various outcomes, change in daily fiber intake was plotted against the percentage relative change of disease outcomes and alpha diversity in Figure 4. When comparing the change of daily intake of fiber to disease-specific outcomes for all disease populations combined, no trend was found (Figure 4A). However, in T2DM patients there was a general negative trend between both FBG and HbA1c and an increase in daily fiber intake (Figure 4B,C). Additionally, a positive association was found between alpha diversity and change in daily fiber intake for all disease populations combined (Figure 4D).

Additionally, to determine if there is a trend between different dietary types and specific changes of the microbiome, an overview of the percentage relative change of individual bacteria per diet type was made (Figure 5A). High-fiber diets resulted in an overall increase in *Akkermanisa*, *Faecalibacterium*, *Bifidobacterium*, *Lactobacillus*, *Roseburia*, and a reduction of pro-inflammatory Proteobacteria. In general, low-fiber diets reduced the beneficial bacteria while supplemental fiber resulted in both positive and negative shifts. When comparing the effects of diet type on alpha diversity, there was a larger increase after high fiber compared to supplemental fiber dietary interventions (+11.68% (two studies, *n* = 17) and +3.33% (four studies, *n* = 108), respectively). Conversely, all diet types showed an overall reduction in fecal SCFA (low fiber −16.98% (two studies, *n* = 33), high fiber −4.55% (four studies, *n* = 66), supplemental fiber −0.84% (two studies, *n* = 66)).

### 3.3. Associations between Microbiome Composition and Disease

A handful of studies reported associations between disease-specific outcomes and changes in the microbiome after dietary intervention in T2DM patients. First, after a macrobiotic dietary intervention, Candela et al. observed a significant inverse association between *Faecalibacterium* and FBG, while *Bacteroides* and *Akkermansia* showed an inverse trend with respect to total and LDL cholesterol [69]. Furthermore, Pedersen et al. showed significant positive associations between unclassified Enterobacteriaceae (Proteobacteria phylum) and FBG and hs-CRP (*p* < 0.05) after fiber supplementation [73]. Additionally, negative correlations were found between alpha diversity (Shannon index) and *B. longum* with HbA1c after high-fiber dietary interventions (rs = −0.458, *p* < 0.05 and *p* < 0.01, respectively) [70,72].

To form a hypothesis regarding whether microbiome changes are a mediatory factor between dietary interventions and observed disease outcomes, Figure 5B compares disease response of the included studies with changes in the microbiome. The studies which showed a significant positive disease response had more beneficial changes of the microbiome compared to the studies with no significant, or a negative, disease response. Specifically, the studies which showed a positive disease response had an increase in alpha diversity while the non-response studies showed a slight reduction (+18.79% (two studies, *n* = 36) and −1.33% (four studies, *n* = 89), respectively). Additionally, *Akkermansia*, *Faecalibacterium*, *Bifidobacterium*, *Lactobacillus* and *Roseburia* all increased in the studies with a positive disease response (Figure 5B). In contrast, the non-response studies had more varying results, whereby on one hand there was a beneficial reduction of Proteobacteria and increase of *Bifidobacterium* and *Roseburia* but also reduction of the beneficial *Faecalibacterium*, *Akkermanisa*, and *Lactobacillus*.

## 4. Discussion

### 4.1. Clinical Benefits Are Mainly Seen with High-Fiber Diets

Dietary interventions with higher fiber intake seem most effective at improving disease-specific outcomes as well as beneficially altering the gut microbiome in patients with chronic inflammatory diseases, especially T2DM. Plant-based dietary interventions such as vegetarian and vegan diets were consistently more effective at improving clinical and microbiome outcomes than other dietary interventions, including Mediterranean dietary patterns. Additionally, increased fiber intake due to a whole diet change caused more beneficial effects than intake of fiber as a supplement. Furthermore, a hypothesis can be formed whereby alterations to the gut microbiome may be a mediating factor in the observed changes of disease-specific outcomes after dietary interventions. Specifically, studies with positive disease response showed a greater increase in SCFA-producing bacteria possibly contributing to the anti-inflammatory effects.

This systematic review highlights the beneficial effect of high-fiber intake for patients with chronic inflammatory diseases. These findings are in line with epidemiological data which show that higher fiber, as well as higher adherence to a plant-based dietary pattern, is associated with a reduction of the risk and incidence of T2DM and CVD [83,84,85,86,87,88]. Overall, fiber intake increases stepwise when moving from healthy omnivorous to plant-based diets [89]. This increase in fiber may help explain the observed clinical benefits of vegan and vegetarian diets on T2DM, CVD, and RA in this systematic review [62,63,68,69]. Interestingly, low-carbohydrate diets, which limit intake of fiber-rich fruits, starchy vegetables, legumes, and whole grains, have been extensively used in the treatment of T2DM [90]. Although these diets have been shown to be effective at inducing T2DM remission after six months compared to other diets, these benefits diminish at 12 months and such diets, when predominantly animal-based, are associated with an increased risk of cardiovascular disease and mortality [90,91].

The Mediterranean diet, a primarily plant-based dietary pattern with an emphasis on healthy fats and small portions of animal-based products, is known as a healthy dietary pattern associated with reduced risk of chronic diseases, such as CVD, and all-cause mortality [92,93]. In this review, Mediterranean diets were only effective at reducing clinical outcomes in patients with T2DM and had a limited or inconsistent clinical effect on patients with RA and IBD [56,64,65,70,71,72]. Additionally, when compared head-to-head, Candela et al. showed that a vegan macrobiotic diet resulted in greater daily fiber intake (+15 g/day) and was more effective at reducing FBG and CRP than a Mediterranean diet [69]. A recent study by Barnard et al. also demonstrated that a low-fat vegan diet was better at reducing metabolic risk factors and insulin resistance compared to a Mediterranean diet [94]. The inconsistent results of Mediterranean diets may be due to greater variations in the interpretation and thus execution of this dietary pattern, whereby some versions may be richer in animal-based products and lower in fiber than others [95].

Of the 11 included studies using fiber supplementation, only two studies showed significant clinical benefits [59,78]. Interestingly, these fiber interventions both consisted of supplementation with whole foods rich in fiber (e.g., germinated barley foodstuff and a functional food supplement containing dehydrated nopal, chia seeds, and inulin) while the other fiber supplements were made up of isolated fibers (e.g., FOS, GOS, inulin, and psyllium). Overall, the high-fiber, whole-diet interventions were more effective at improving disease-specific outcomes than supplementation. Consequently, nutrients other than fiber could contribute to the clinical benefits of the high-fiber diets. On the other hand, King et al. showed that a 25–30 g/day psyllium fiber supplementation was just as effective as a high-fiber diet at reducing CRP levels [12]. The included studies may therefore not have supplemented with high enough dosages (ranging from 5.5 to 16.5 g/day) to show effect. Furthermore, different types of fiber have varying biological effects. Gel-forming fiber (e.g., beta-glucans, psyllium) improves glycemic control and lowers serum cholesterol, while other types of fiber, such as insoluble fiber (e.g., wheat bran) or soluble, non-viscous fiber (e.g., inulin, wheat dextrin, oligosaccharides, and resistant starches), do not carry these properties [96]. A diverse whole-food dietary intervention with a mixture of types of fiber may thus also contribute to the clinical effects observed.

Although not included in this review, more literature is available on the effect of dietary interventions in chronic disease in studies that did not evaluate microbiome composition changes. For RA, T2DM, IBD, and CVD, Mediterranean and other plant-based diets have been shown to reduce clinical outcomes [97,98,99,100,101,102,103]. For IBD, there are also numerous studies showing the benefits of low FODMAP dietary interventions [101].

### 4.2. High-fiber Diets Can Increase Microbial Diversity and SCFA-Producing Bacteria

In general, high-fiber whole-diet interventions resulted in more beneficial microbiome outcomes compared to both low-fiber diets and fiber supplements. Overall, 70% of the high-fiber dietary interventions showed a beneficial shift of the microbiome by increasing diversity, increasing the abundance of SCFA-producing bacteria, and/or decreasing pathogenic bacteria [56,57,58,62,63,66,67,69,70]. Of the fiber supplement interventions, six of the 11 studies showed an overall beneficial shift of the microbiome, of which three were synbiotic interventions [59,60,75,76,78,79,81]. Both low-FODMAP studies negatively affected the microbiome, while two other low-fiber diets showed some beneficial shifts [52,53,54,55]. Overall, these findings are congruent with other studies which show high-fiber and low-fat plant-based diets can increase the abundance of beneficial polysaccharide-digesting bacterial species [41,104]. However, considering the known prebiotic effects of the various fiber supplements used in the included trials (e.g., GOS, FOS, and psyllium), a beneficial change in microbiome would have been expected in these studies as well [43]. Variations in baseline individual microbiome composition may be a determining factor of whether individuals respond to a dietary intervention or fiber supplement [105]. Additionally, other factors such as age, exercise, stress, and environment, as well as other dietary components such as fat and protein intake, can also alter the microbiome and thus potentially influence the results of the included studies [41,106].

Regardless of fiber intake, the included studies which measured SCFAs showed an overall decrease in SCFA levels. Although these results are unexpected, measuring fecal concentrations of SCFAs, as was done in these studies, is an inaccurate method [43,107]. This is because, amongst other factors, SCFAs are absorbed in the gut and thus the rate of SCFA production cannot be accurately estimated [107]. This review also showed that studies with significant clinical response had increased alpha diversity and a trend towards more beneficial changes of the microbiome compared to studies with no (significant) clinical benefits. Consequently, it can be hypothesized that the observed increase of SCFA-producing bacteria may have played a role in reducing inflammation and thus mediating the observed improvements in disease-specific outcomes. However, these observations are merely trends and must be further substantiated in future research. Other metabolites secreted by microorganisms that cause a beneficial effect to the host, also known as postbiotics, can potentially play an anti-inflammatory role [108]. Although this review mainly focused on SCFAs, future research should consider other postbiotics and their potential mediatory role between microbiome and host health.

One’s core microbiome is shaped early in life (from 4 to 36 months) and from two–three years of age is considered relatively stable yet susceptible to changes [16]. As a result, the relatively short duration of the included studies (median 12 weeks (range 2–107 weeks)) can only indicate short-term effects on the microbiome. Whether the dietary interventions can cause permanent changes to the relatively resilient adult microbiome cannot be determined based on these studies.

### 4.3. Methodological Considerations

This systematic review has various strengths. First, it provides a holistic view of chronic inflammatory disorders, allowing for a further understanding of effects of dietary interventions on the shared pathophysiology of the included disease populations. By comparing the studies based on relative change of disease-specific outcomes, a general impression can be made about how different dietary interventions influence chronic inflammatory diseases, which otherwise may go unnoticed. Similarly, by sorting the studies into dietary categories by fiber intake it also allows for an overview of the effects of different types of fiber interventions. Although no correlations can be found, by stratifying the studies into “responder” and “non-responder” groups, a hypothesis about the potential mediatory effects of the microbiome is formed.

Despite the attempt to create a holistic view of chronic inflammatory diseases, it remains difficult to compare studies due to the different disease-specific outcomes used and disease characteristics. Although inflammation plays a role in the pathophysiology of the included disease types, the included disease types have different disease properties. For example, T2DM is a metabolic condition with inflammatory components, while IBD and RA are inflammatory autoimmune disorders. Using the described methodology in this review, a general hypothesis can be made about potential beneficial dietary patterns for chronic inflammatory disorders but comparing disease types with each other one-on-one is currently not possible.

This heterogeneity in outcomes is not limited to disease types, but also within IBD studies; for example, a large range of outcomes were used to quantify disease activity. This makes it difficult to accurately compare the outcomes of these studies and the clinical significance of the percentage relative changes shown in Figure 3 and Figure 4. Since medications influence the microbiome in different ways and many different medications were used in the trials, there is a further need to interpret the outcomes with caution [109,110]. Furthermore, short-term fiber supplementation may increase gastro-intestinal symptoms and disease activity in IBD patients, thus increasing the difficulty of comparing results of fiber interventions in IBD patients to other diseases [60,61]. This could explain why in Figure 4A no trend was seen between the change in daily fiber intake and disease-specific outcome for all diseases combined, yet when isolating T2DM patients in Figure 4B,C higher fiber intakes led to decreased FBG and HbA1c. Consequently, in IBD patients, high-fiber diets where fiber is introduced gradually may be most beneficial as it allows for the gut microbiome to adjust, thus reducing abdominal complaints. Considering low-fiber diets, such as a low-FODMAP, negatively impacted the microbiome without improving disease activity, for IBD patients these diets should be used with caution [52,53]. Lastly, differences in group size between studies must be considered as a limitation. In total, 62% of the included studies had <50 enrolled participants, 27% had 50–100 participants, and 10% >100 participants. Specifically, small study populations may be inadequate for microbiome analyses due to the large inter-individual variation of the microbiome [111].

Due to the heterogeneity of the studies, only trends can be observed between dietary interventions, disease-specific outcomes, and microbiome outcomes when comparing studies. Furthermore, although Figure 3, Figure 4 and Figure 5 provide information about potential associations between variables, the data must be interpreted with caution and can merely be used to form additional hypothesis and inspire additional research.

To narrow the scope of this study, it was chosen to only include dietary interventions with whole diet interventions or fiber supplements. However, it is known that other dietary components, such as protein and fat, can also influence the microbiome [40,44,98]. Moreover, not all studies reported the changes of individual dietary components, such as fiber, making it difficult to accurately compare the dietary interventions.

Lastly, the microbiome scientific field is relatively young and quickly evolving. As a result, there are various limitations to the methods used in microbiome research [105]. Currently, 16S ribosomal RNA (16S rRNA) sequencing is the most used method for analyzing bacterial species. Overall, this is an advanced method, especially compared to gas-liquid chromatography or FISH. However, this technique has inaccuracies at species-level classification, and thus in the future microbiome research should focus on using whole metagenome sequencing techniques [112].

In future studies, to gain a greater understanding of the effect of dietary interventions on chronic inflammatory diseases, a holistic view including various diseases is recommended. To accomplish this more accurately, standardized outcomes should be used to quantify disease-specific outcomes and dietary assessments should be used to supply data on changes in nutrient intake. More dietary intervention studies are also necessary in chronic inflammatory diseases (e.g., IBD, RA, and CVD) to evaluate if the observed trends are indeed applicable for chronic inflammatory diseases as a whole or are rather disease specific.

## Figures and Tables

**Table 1:** Characteristics of the included studies and overview of outcomes in patients with: (a) inflammatory bowel disease, (b) rheumatoid arthritis, (c) cardiovascular disease, (d) type 2 diabetes.

First Author, Publication Year, Journal	Disease and Number of Cases (*n =* Enrolled (# of Dropouts))	Study Design and Duration	Type of Intervention	Disease-Specific Outcome Baseline		Disease-Specific Outcome End Intervention		Change	Remarks	Gut Microbiome End Intervention	
				Intervention	Control	Intervention	Control	*p*-Value		Change	Remarks
**(a) Inflammatory Bowel Disease**											
Walters 2014 SOJ Microbiology and Infectious Diseases [54]	Crohn’s disease (*n* = 6 (2))	Double-blinded cross-over RCT 12 weeks (4 weeks per intervention, 4-week wash-out)	*Low fiber* Specific carbohydrate diet (SCD) vs. low residue diet (LRD)	-	-	-	-	-	No numerical results. No significant clinical improvement in IBD patients receiving the SCD diet.	↑ Diversity A general increase in diversity was observed mainly due to an increase of non-pathogenic clostridia species in SCD vs. LRD groups.	
Halmos 2016 Clinical and Translational Gastroenterology [53]	Crohn’s disease (*n* = 9)	Single-blinded (participant) cross-over RCT 9 weeks (3 weeks per intervention, 3-week wash-out)	*Low fiber* Low FODMAP diet with 8 g/day prebiotic vs. typical (Australian) diet	-	-	-	-	-	No numerical results. Figure 1 showed no observable difference in FC between groups.	↔ SCFA *↑ Ruminococcus torques* ↔ *Bifidobacterium* ↓ *Clostridium coccoides* group *↓ A. municinphilla* ↔ *F. prausnitzii*	8 participants collected fecal samples and *n* = 7 for microbial analysis of *A. muciniphila*.
Cox 2020 Gastroenterology [52]	Ulcerative colitis and Crohn’s disease (*n* = 52 (6))	Single-blinded (participant) RCT 4 weeks	*Low fiber* Low FODMAP diet vs. Sham diet	FC (μg/g): 54.8 (84.8)	FC: 70.9 (117.3)	PMS: 0.2 [0.2] HBIS: 3.2 [0.4] FC: 53.3 (84.8) CRP (mg/L): 2.0 [0.3]	PMS: 0.2 [0.2] HBIS: 3.4 [0.5] FC: 66.9 (106.4) CRP: 1.6 [0.3]	*p* = 0.951 *p* = 0.841 *p* = 0.976 *p* = 0.246	*p*-value of FC based on intervention (60.0 [9.4]) vs. control (59.6 [9.8] (μg/g))	↔Alpha diversity ↔ Total SCFA ↔ Total *Bifidobacterium* *↓ B. Adolescentis* *↓ B. Longum* *↑ B. Dentium* *↓ F. Prausnitzii*	Micobiome outcomes measured with quantitative metagenomic pipeline. Alpha diversity measured using Shannon index. Outcomes reported per protocol (*n* = 43).
Marlow 2013 Human Genomics [56]	Crohn’s disease (*n* = 8)	Uncontrolled trial 6 weeks	*High fiber* Mediterranean-inspired anti-inflammatory diet	-	NA	-	NA	-	No numerical results, CRP showed a trend of reduction (*p* = 0.39).	*Clostridium leptum* group and Bacteroidetes increased. Proteobacteria and Bacillaceae decreased.	No statistical analysis
Fritsch 2020 Clinical Gastroenterology and Hepatology [57]	Ulcerative colitis (*n* = 26 (9))	Nonblinded cross-over RCT 10 weeks (4 weeks per intervention, 2-week wash-out)	*High fiber* Low fat, high fiber diet (LFD) vs. improved standard American diet (iSAD)	PMS: 1.41 [2] FC (μg/g): 88.7 (149) CRP (mg/L): 3.23 (3.88)	PMS: 1.41 [2] FC: 88.7 (149) CRP: 3.23 (3.88)	PMS: 0.6 [1.5] FC: 66.16 (61.52) CRP: 2.51 (1.61)	PMS: 0.76 [2] FC: 124.8 (141.5) CRP: 3.05 (2.92)	*p* = 0.63 *p* = 0.19 *p* = 0.21		↔ Alpha diversity *↑ F. Prausnitzii* ↔ *Prevotella* Within LFD: ↔ Alpha diversity ↔ *F. Prausnitzii* ↑ *Prevotella* Within iSAD: ↔ Alpha diversity ↔ *F. Prausnitzii* ↔ *Prevotella*	Alpha diversity measured using Faith’s phylogenic diversity index
Zhang 2020 Journal of Crohn’s and Colitis [58]	Crohn’s disease (*n* = 58 (18))	Nonblinded non-randomized trial 12 weeks	*High fiber* non-diversified diet (NDD) group (Mediterranean-inspired diet) vs. diversified diet (DD) group (conventional management)	FC (μg/mg): 159.08 ± 277.9	-	FC: 178.45 ± 224.1	FC: 115.45 ± 159.4	*p* = 0.56	Within NDD group no significant difference baseline vs. end (*p* = 0.26)	↔ Alpha diversity ↔ Beta diversity ↔ *Faecalibacterium* ↓ Proteobacteria ↓ *Escherichia/Shigella* Within NDD group: ↔ SCFA ↑ *Faecalibacterium* ↔ Proteobacteria ↔ *Escherichia/Shigella* ↔ *Bifidobacterium* ↔ *Akkermansia*	Alpha diversity measured using Shannon Index. Beta diversity measured using Manhattan and Gower distance matrices between bacterial communities.
Kanauchi 2002 Journal of Gastroenterology [59]	Ulcerative colitis (*n* = 18)	Single-blinded (observer) RCT 4 weeks	*Supplemental fiber* 20-30 g/day germinated barley foodstuff (GBF) with baseline medical treatment vs. baseline medical treatment	CAI (Lichtiger method): 8 [5] CRP (ng/mL): 0.96 [1.75]	CAI: 7 [4] CRP: 0.52 [0.63]	CAI: 6 [3] CRP: 0.6 [1]	CAI: 10 [5] CRP: 0.5 [0.5]	*p* = 0.045 *N.S.*	Numerical results estimated from Figure 2 for CAI and from Figure 3 for end of intervention CRP.	Within GBF group: Increased *Bifidobacterium*, *Eubacterium limosum*, and *Lactobacillus*. Decreased *Bacteroides*.	Microbiome outcomes only reported for intervention group and statistical significance not reported.
Furrie 2005 Gut [60]	Ulcerative colitis (*n* = 16 (4))	Double-blinded RCT 4 weeks	*Supplemental fiber* 12 g/day of inulin-ogliofructose with *Bifidobacterium longum* synbiotic per day vs. placebo	CAI (Walmsley method): 5.6 ± 3.7 CRP (mg/L): 6.0 ± 6.5	CAI: start 4.9 ± 3.2 CRP: 1.6 ± 3.6	CAI: 5.3 ± 3.4 CRP: 1.8 ± 3.9	CAI: 3.1 ± 2.5 -	Not reported.	No numerical results for CRP of control group at end intervention, but authors reported that none of the control patients had circulating levels of CRP.	Within prebiotic group: 42-fold increase in *Bifidobacterium* Within placebo group: 4.6-fold increase in *Bifidobacterium*	Microbiome outcomes measured using real time PCR in mucosal biopsies. Only within group outcomes reported, no statistical analysis was performed.
Benjamin 2011 Gut [61]	Crohn’s disease (*n* = 103 (18))	Double-blinded RCT 4 weeks	*Supplemental fiber* 15 g/day fructo-oligosaccharides (FOS) vs. placebo	CDAI: 283 ± 61 FC (mg/kg): 621.4 ± 559.4 CRP (mg/L): 18.8 ± 21.6	CDAI: 286 ± 62 FC: 647.9 ± 533.3 CRP: 20.8 ± 23.1	CDAI: 250 ± 84.9 FC: 657.4 ± 592.5 CRP: 20.9 ± 26.4	CDAI: 220 ± 88.7 FC: 829.8 ± 635.9 CRP: 20.2 ± 24.1	*p* = 0.112 *p* = 0.322 *p* = 0.902	FC reported per protocol (*n* = 60).	↔ *F. Prausnitzii* ↔ *Bifidobacterium*	Microbiome outcomes measured with FISH technique.
**(b) Rheumatoid Arthritis**											
Peltonen 1994 British Journal of Rheumatology [62]	Rheumatoid arthritis (*n* = 53 (19))	Single-blinded (observer) RCT 56 weeks	*High fiber* 7 day fast followed by 1-year vegetarian diet (first 3.5 months vegan) vs. ordinary omnivore diet	DAS28†: 5.3 CRP (mg/L): 23	DAS28: 4.7 CRP: 23	DAS28: 3.6 CRP: 19	DAS28: 5.5 CRP: 30	- *p* = 0.0001	DAS28 estimated from Figure 1 and Figure 2, CRP estimated from Figure 1. All components used in DAS28 were significantly lower in the intervention group vs. control at end intervention.	Significant difference in CFA profiles between baseline samples and end of intervention.	Microbiome outcomes measured using direct gas-liquid chromatography (GLC) to measure bacterial cellular fatty acid (CFA) profiles of stool samples.
Peltonen 1997 British Journal of Rheumatology [63]	Rheumatoid arthritis (*n* = 43 (7))	Single-blinded (observer) RCT 4 weeks	*High fiber* Raw vegan diet vs. ordinary omnivore diet	-	-	RADII: 3.1	RADII: 2.0	*p* = 0.027	No baseline results reported.	The raw vegan group had a significantly lower similarity index to the baseline samples than the control group.	Microbiome outcomes measured using direct gas-liquid chromatography (GLC) to measure bacterial cellular fatty acid (CFA) profiles of stool samples.
Michalsen 2005 BMC Complementary and alternative medicine [64]	Rheumatoid arthritis (*n* = 16)	Nonblinded non-randomized trial 2 weeks	*High fiber* Mediterranean-inspired (Med) diet vs. fasting	DAS28: 5.2 ± 1.9	DAS28: 5.2 ± 2.0	DAS28: 5.0 ± 2.1	DAS28: 4.5 ± 2.0*	*p* = 0.09	Results estimated from Figure 2.	Within both Med diet and fasting groups: ↔ E. coli ↔ *Enterococcus* ↔ *Lactobacillus* ↔ *Clostridium* ↔ *Bifidobacterium*	No results reported for between groups.
Abendroth 2010 Forschende Komplementarmedizin [65]	Rheumatoid arthritis (*n* = 50)	Nonblinded non-randomized trial 2 weeks	*High fiber* Mediterranean-inspired (Med) diet vs. fasting	DAS28: 5.4 ± 1.4 CRP (mg/L): 20 ± 27	DAS28: 5.6 ± 1.1 CRP: 8 ± 1	DAS28: 4.6 ± 1.1 * CRP: 16 ± 22	DAS28: 4.1 ± 1.3 * CRP: 7 ± 7	*p* = 0.115 *p* = 0.59	Results estimated from Figure 1.	↔ SCFA Within Med diet group: SCFA: 22.9 ± 13.8 to 20.4 ± 9.8 Within Fasting group: SCFA: 26.5 ± 12.9 to 30.0 ± 23.9	Within group results were not statistically analyzed
**(c) Cardiovascular Disease**											
Haro 2016 Journal of Clinical Endocrinology and Metabolism [66]	Cardiovascular disease (*n* = 20)	Single-blinded (observer) RCT 52 weeks	*High fiber* Low-fat high complex carbohydrate diet vs. Mediterranean-inspired diet	LDL (mg/dL): 83.7 [5.7] HDL (mg/dL): 40.3 [2.4] TC (mg/dL): 149.8 [6.9] TG (mg/dL): 102.2 [7.9]	LDL: 88.0 [5.7] HDL: 42.1 [2.4] TC: 150.2 [6.9] TG: 98.7 [7.9]	LDL: 81.0 [5.1] HDL: 39.7 [2.0] TC: 142.2 [5.8] TG: 104.8 [11.5]	LDL: 85.9 [5.1] HDL: 44.8 [2.0] TC: 148.2 [5.8] TG: 85.8 [11.5]	*p* = 0.917 *p* = 0.250 *p* = 0.501 *p* = 0.313	No numerical results available for SBP, no significant difference was found. No significant differences were found within the groups between baseline and end.	↔ Alpha diversity ↔ *F. prausnitzii* *↑ Prevotella* Within low-fat diet group: *↑ F. prausnitzii* *↓ Roseburia* Within Mediterranean diet group: *↑ Roseburia*	
Haro 2017 Molecular Nutrition and Food Research [67]	Cardiovascular disease (*n* = 33)	Single-blinded (observer) RCT 104 weeks	*High fiber* Low-fat high complex carbohydrate diet vs. Mediterranean-inspired diet	-	-	-	-	-	No numerical results. Both diets lowered TG levels (*p* <0.001). CRP was reported together for the two diet groups. Compared to baseline there was no significant change in CRP.	Within both low-fat and Mediterranean diet groups: ↑ *Bacteroides* ↑ *Prevotella* ↑ *Faecalibacterium*	No results reported for between groups.
Djekic 2020 Journal of the American Heart Association [68]	Cardiovascular disease (*n* = 31 (4))	Nonblinded cross-over RCT 12 weeks	*High fiber* Vegetarian diet vs. conventional Swedish diet	LDL (mg/dL): 61.9 (55.7–68.4) HDL (mg/dL): 47.6 (42.9–53.0) TC (mg/dL): 134.6 (124.9–144.2) TG (mg/dL): 86.8 (76.2–98.3) SBP (mmHg): 136 (129–143) CRP (mg/L): 0.73 (0.51–1.03)	LDL: 63.8 (58.0–69.6) HDL: 49.1 (44.5–54.1) TC: 136.9 (129.9–145.0) TG: 87.7 (77.1– 99.2) SBP: 140 (133–146) CRP: 0.81 (0.60–1.09)	LDL: 54.5 (49.5–59.6) * HDL: 44.5 (39.8–49.9) * TC: 124.1 (116.00–131.9) * TG: 92.1 (83.3–102.7) SBP: 133 (127–140) CRP: 0.74 (0.50–1.09)	LDL: 58.8 (52.6–65.0) * HDL: 46.1 (41.4–51.43)* TC: 129.2 (120.6–137.6)* TG: 86.8 (77.1–98.3) SBP: 136 (129–142) CRP: 0.81 (0.55–1.18)	*p* = 0.02 *p* = 0.2 *p* = 0.01 *p* = 0.1 *p* = 0.1 *p* = 0.6		↔ Alpha diversity ↔ Beta diversity ↔ Total SCFA ↔ *Lactobacillus* ↔ *Bacteroides* ↔ *Roseburia* ↔ *Collinsella* ↔ *Bifidobacterium* ↔ *Faecalibacterium* *↑ Akkermansia*	Microbiome outcomes only available for *n* = 20.
**(d) Type 2 Diabetes**											
Ren 2020 Nutrients [55]	Type 2 diabetes (*n* = 50) (5))	Nonblinded RCT 12 weeks	*Low fiber* Low-carb diet with 56 g/day almonds vs. low-fat diet	HbA1c (%): 7.64 ± 1.50	HbA1c: 7.54 ± 1.25	HbA1c: 6.91 ± 1.00 *	HbA1c: 7.38 ± 1.24*	*p* <0.01		↔ Alpha diversity ↔ Beta diversity ↑ *Roseburia* ↓ *Eubacterium* ↑ *Ruminococcus* ↔ *Lactobacillus* ↔ *Bacteroides* Within low-carb diet group: ↑ *Eubacterium* ↓ *Bacteroides* *↑ Roseburia* Within low-fat diet group: ↓ *Ruminococcus* ↓ *Roseburia*	
Candela 2016 British Journal of Nutrition [69]	Type 2 diabetes (*n* = 40)	Single-blinded (observer) RCT 3 weeks	*High fiber* Macrobiotic (Ma-Pi 2) diet vs. Mediterranean-inspired (Med) diet	FBG (mg/dL): 126 (43) HbA1c (%): 6.5 (1.6) CRP (mg/L): 3.2 (10.6)	FBG: 138 (42) HbA1c: 6.9 (1.1) CRP: 2.7 (4.5)	FBG: 95 (15)* HbA1C: 6.1 (1.2) CRP: 1.0 (1.8) *	FBG: 108 (12.5)* HbA1C: 6.8 (0.9) CRP: 1.6 (3.8)	*p* <0.0002 *p* = 0.3 *p* = 0.03		↔ Beta diversity Within both Ma-Pi 2 and Mediterranean diet group: ↔ Alpha diversity	Bacterial outcomes reported as deviation in terms of fold change from the median profile of healthy subjects (Figure 4). Statistical significance not reported.
Zhao 2018 Science [70]	Type 2 diabetes (*n* = 49) (6))	Nonblinded RCT 12 weeks	*High fiber* High-fiber diet with 600 mg/day acarbose vs. usual care with 600 mg/day acarbose	FBG (mmol/L): 8.47 [0.42] HbA1c (%): 8.27 [0.27]	FBG: 8.91 [0.57] HbA1c: 8.31 [0.38]	FBG: 6.37 [0.20]* HbA1c: 6.36 [0.11]*	FBG: 6.92 [0.47]* HbA1c: 7.01 [0.27]*	*p* = 0.2233 *p* = 0.0122		↑ Beta diversity ↔ Total SCFA Higher abundance of *F. prausnitzii* and *Eubacterium rectale*, less *Lactobacillus reuteri* and *Bifidobacterium longum.*	Statistical significance not reported for bacteria species outcomes.
Liu 2020 Experimental and Therapeutic Medicine [71]	Type 2 diabetes (*n* = 140) (124))	Uncontrolled trial 26 weeks	*High fiber* Mediterranean-inspired diet	FBG (mmol/L): 8.2 HbA1c (mmol/L): 6.9	NA	FBG: 7.1 HbA1c: 6.3	NA	*p* <0.001 *p* <0.001	Results estimated from Figure 2.	Within Med diet group: ↔ *Roseburia* ↔ *Lachnospira* ↑ *Pseudomonas*	
Ismael 2021 Nutrients [72]	Type 2 diabetes (*n* = 11) (3))	Uncontrolled trial 12 weeks	*High fiber* Personalized Mediterranean diet based on participant’s dietary history and nutritional needs	FBG (mg/dL): 131.63 ± 8.53 HbA1c (%): 7.53 ± 1.07 CRP (mg/L): 2.0 ± 2.0	NA	FBG: 122.50 ± 9.42 HbA1c: 6.86 ± 0.85 CRP: 2.5 ± 3.3	NA	*p* = 0.581 *p* = 0.024 *p* = 0.21		↔ Alpha diversity ↔ Firmicutes: Bacteroidetes ↔ Prevotella: Bacteroides Increase of *Bacteroides*, *Ruminococcus*, *Akkermansia*, *Roseburia* and decrease of *Faecalibacterium* and *Prevotella.*	Statistical significance not reported for bacteria genus and species outcomes.
Pedersen 2016 British Journal of Nutrition [73]	Type 2 diabetes (*n* = 32) (3))	Double-blinded RCT 12 weeks	*Supplemental fiber* 5.5 g/day prebiotic fiber (GOS mixture) vs. placebo	FBG (mmol/L): 6.1 [0.4] HbA1c (mmol/mol): 51.2 [3.1] CRP (mg/L): 1.31 (0.97)	FBG: 6.2 [0.3] HbA1c: 46.3 [1.8] CRP: 1.65 (3.13)	FBG: 6.8 [0.4]* HbA1c: 53.1 [3.2] CRP: 1.26 (2.36)	FBG: 6.5 [0.3] HbA1c: 48.4 [2.4] CRP: 0.92 (1.37)	*p* = 0.227 *p* = 0.946 *p* = 0.444		↔ Alpha diversity ↔ *Bifidobacterium* ↔ *Lactobacillus* ↔ *Roseburia* ↔ *Enterobacteriacea* Within prebiotic group: ↑ Alpha diversity	
Gonai 2017 Beneficial Microbes [74]	Type 2 diabetes (*n* = 55 (3))	Double-blinded RCT 4 weeks	*Supplemental fiber* 10 g/day galacto-oligosaccharides (GOS) vs. placebo	FBG (mg/dL): 132.3 ± 26.1 HbA1c (%): 7.1 ± 1.2	FBG 130.0 ± 27.3 HbA1c: 6.8 ± 0.9	FBG 138.4 ± 31.0 HbA1c: 7.0 ± 1.1	FBG:138.2 ± 33.6 HbA1c: 6.8 ± 1.1	-	No significant differences were found.	↓Alpha diversity ↑ Bifidobacteriacea ↔ Ruminococcacea ↓ Lachnospiraceae Within GOS group: ↓ Alpha diversity ↑ Bifidobacteriacea ↓ Ruminococcacea ↓ Lachnospiraceae Within placebo group: ↔ Alpha diversity ↔ Bifidobacteriacea ↔ Ruminococcacea	
Roshanravan 2017 Journal of Cardiovascular and Thoracic Research [75] Roshanravan 2018 European Journal of Integrative Medicine [76]	Type 2 diabetes (*n* = 30)	Double-blinded RCT 6.5 weeks	*Supplemental fiber* 10 g/day inulin vs. placebo	FBG (mg/dL): 167.07 ± 82.17 CRP (mg/L): 5.45 ± 2.28	FBG: 129.53 ± 26.38 CRP: 5.40 ± 2.01	- CRP: 3.80 ± 1.38*	- CRP: 5.91 ± 2.15*	*p* = 0.309 *p* <0.001	No numerical results reported for end intervention, change from baseline levels depicted in Figure 4.	*↑ A. muciniphilia* Within inulin group: *↑ A. muciniphilia* Within placebo group: ↔ *A. muciniphilia*	
Lee 2019 Diabetes and Metabolism Journal [77]	Type 2 diabetes (*n* = 14) (4))	Uncontrolled trial 4 weeks	*Supplemental fiber* 12 g/day psyllium supplement	QUICKI§: 0.34 (0.06)	NA	QUICKI: 0.31 (0.05)	NA	*p* >0.05		*↓ Blautia* *↓ Blautia wexlerae* *↓ Eubacterium* *↓ Bifidobacterium longum* *↓ Enterobacter soli*	
Medina-Vera 2019 Diabetes and Metabolism [78]	Type 2 diabetes (*n* = 81) (28))	Double-blinded RCT 12 weeks	*Supplemental fiber* 16.5 g/day fiber in high-fiber functional food supplement with reduced calorie diet vs. reduced calorie diet	HbA1c (%): 7.5 ± 1.3	HbA1c: 6.9 ± 1	-	-	*p* <0.05 *p* <0.01	End intervention −7.20% difference of HbA1c and −13% difference CRP between groups.	↑ Alpha diversity *F. prausnitzii* +34%, *A. muciniphilia* +125%, *P. copri* −13%, *B. longum* and *B. fragilis* increased.	Statistical significance not reported for bacteria species outcomes.
Horvath 2020 European Journal of Nutrition [79]	Type 2 diabetes (*n* = 26 (15))	Double-blinded RCT 26 weeks	*Supplemental fiber* 8/g day GOS and FOS with *Lactobacillus* and *Bifidobacterium* spp. synbiotic vs. placebo	FBG (mg/dL): 177 (147–207) HbA1c (mmol/mol): 64 (53–74)	FBG: 174 (148–200) HbA1c: 62 (59–66)	FBG: 188 (142–235) HbA1c: 67 (54–80)	FBG: 163 (134–191) HbA1c: 64 (58–71)	*p* = 0.5 *p* = 0.8	No statistical analysis performed within groups.	↔ Alpha diversity ↔ Beta diversity Within both prebiotic and placebo group: ↔ Alpha diversity ↔ Beta diversity	
Reimer 2021 European Journal of Nutrition [80]	Type 2 diabetes (*n* = 290) (190))	Double-blinded RCT 52 weeks	*Supplemental fiber* 15 g/day highly viscous polysaccharides with low calorie diet vs. placebo with low calorie diet	FBG (mmol/L): 7.4 ± 1.9 HbA1c (%): 7.2 ± 1.1	FBG: 7.3 ± 1.9 HbA1c: 7.0 ± 0.9	FBG: 7.59 HbA1c: 6.97*	FBG: 7.71 HbA1c: 6.96	*p* = 0.955 *p* = 0.516		↔ Alpha diversity ↔ Beta diversity *↓ F. prausnitzii* ↔ *A. muciniphila* ↔ *Roseburia* ↓ *Lactobacillus* ↑ *Collinsella*	*n* = 47 for diversity outcomes, *n* = 87 for microbial outcomes.
Kanazawa 2021 Nutrients [81]	Type 2 diabetes (*n* = 88 (8))	Nonblinded RCT 24 weeks	*Supplemental fiber* 7.5 g/day GOS with *Lactobacillus parasei* and *Bifidobacterium breve* synbiotic vs. no synbiotic	FBG (mg/dL): 140.5 ± 3 3.6 HbA1c (%): 7.4 ± 0.7 CRP (mg/dl): 603.5 (1515.5)	FBG: 131.7 ± 21.5 HbA1c: 7.3 ± 0.8 CRP: 1050 (1350)	FBG: 146.7 ± 41.1 HbA1c: 7.6 ± 1.0 CRP: 743.5 (1479)	FBG: 135.2 ± 29.9 HbA1c: 7.4 ± 0.8 CRP: 819 (1996)	*N.S.* *N.S.* *N.S.*		↔ Alpha diversity ↔ Total SCFA ↑ *A. muciniphilia* ↔ *Prevotella* *↑ Lactobacillus* *↑ Bifidobacterium* ↔ Proteobacteria Within synbiotic group: ↔ Alpha diversity ↑ Total SCFA ↔ *A. muciniphilia* ↑ *Prevotella* ↑ *Lactobacillus* ↑ *Bifidobacterium* ↓ Proteobacteria	No statistically significant changes between baseline and end intervention within the control group.

Disease-specific outcomes and CRP shown as: (mean ± SD, mean (SE), mean (95% CI lower range—95% CI upper range) or median (IQR)). CDAI = Crohn’s Disease Activity Index, CAI = Clinical activity index, PMS = Partial Mayo score for UC, HBIS = Harvey-Bradshaw index score for CD, FC = Fecal calprotectin, DAS28 = Disease activity score of Rheumatoid arthritis, ESR = Erythrocyte sedimentation rate, RADII = RA Disease improvement index, LDL = Low-density lipoprotein cholesterol, HDL = High-density lipoprotein, TC = Total cholesterol, Tg = Triglycerides, SBP = Systolic blood pressure, FBG = Fasting blood glucose, HbA1c = Hemoglobin A1c, QUICKI = Quantitative insulin sensitivity check index, CRP = C-reactive protein, SCFA = Short-chain fatty acids. *p*-Values represent between group statistical analysis at the end of the intervention, for uncontrolled trials *p*-values represent statistical analysis between baseline and end of the trial. * = Significant difference (*p* < 0.05) within groups baseline vs. end of intervention. † = DAS28 is estimated based on ESR, number of tender and swollen joints, and visual analogue scale (VAS) pain from Figure 1 and Figure 2 from Kjeldsen et al. [82]. § = The quantitative insulin sensitivity check index (QUICKI) is calculated using the inverse of the sum of the logarithms of the fasting insulin and fasting glucose: 1/(log(fasting insulin μU/mL) + log(fasting glucose mg/dL)), lower numbers reflect greater insulin resistance. Microbiome results are shown as a significant (*p* < 0.05) increase (↑), decrease (↓) or no significant difference (↔). Changes in gut microbiome, unless otherwise mentioned, are reported for between groups whereby arrows indicate effect of intervention in relation to control when applicable. Microbiome bacteria are quantified using relative abundance (%). 16s rRNA sequencing used to quantify microbiome outcomes unless otherwise mentioned. SCFA was measured in feces. *N.S*. = Not statistically significant.

## Supplementary Materials

The following are available online at https://www.mdpi.com/article/10.3390/nu13093208/s1, Supplement S1: Search strategy, Figures S1–S3: Risk of bias assessment.
