# Supplementary material for: The Effect of Dietary Interventions on Chronic Inflammatory Diseases in Relation to the Microbiome: A Systematic Review
Source: Nutrients. 2021 Sep 15;13(9):3208. doi: 10.3390/nu13093208 (PMC8464906; doi:10.3390/nu13093208)
Supplement: Supplementary file 1 [file nutrients-13-03208-s001.zip › nutrients-1375577-supplementary.pdf]

Supplement 1: Search strategy

PubMed Session Results 5 January 2021
[Mesh terms] = Medical subject headings
[Mesh:NoExp] = Medical subject headings without explosion
[tiab] = words in title or abstract or author keywords

#	QUERY	RESULTS
**#6**	#5 AND ("Clinical Trial" [Publication Type] OR "Clinical Trials as Topic"[Mesh] OR randomi*[tiab] OR randomly[tiab] OR trial[tiab] OR controls[tiab] OR “control group”[tiab] OR “controlled study”[tiab])	1,207
**#5**	#4 NOT (“Animals”[Mesh] NOT “Humans”[Mesh])	6,420
**#4**	#1 AND #2 AND #3	8,178
**#3**	“Autoimmune Diseases”[MeSH Terms] OR “Arthritis, Rheumatoid”[Mesh] OR “lupus erythematosus, systemic”[MeSH Terms] OR “arthritis, reactive”[MeSH Terms] OR “colitis, ulcerative”[MeSH Terms] OR “Inflammatory Bowel Diseases”[MeSH Terms] OR “Crohn Disease”[MeSH Terms] OR “Myositis”[MeSH Terms] OR “Sarcoidosis”[MeSH Terms] OR “Behcet Syndrome”[MeSH Terms] OR “spondylitis, ankylosing”[MeSH Terms] OR “Psoriasis”[Mesh] OR Autoimmune disease*[tiab] OR Arthritides*[tiab] OR Polyarthritis[tiab] OR Polyarthritides[tiab] OR inflammatory bowel disease*[tiab] OR ulcerative colitis[tiab] OR crohn’s disease*[tiab] OR Crohn's Enteritis[tiab] OR Crohns Disease*[tiab] OR Granulomatous Enteritis[tiab] OR Ileocolitis[tiab] OR Colitis, Granulomatous[tiab] OR Granulomatous Colitis[tiab] OR Terminal Ileitis[tiab] OR Regional Ileitis[tiab] OR Idiopathic Proctocolitis[tiab] OR Ulcerative Colitis[tiab] OR Colitis Gravis[tiab] OR “Osteoarthritis”[Mesh:NoExp] OR “Osteoarthritis, Hip”[Mesh] OR “Osteoarthritis, Knee”[Mesh] OR Coxarthros*[tiab] OR Osteoarthritides[tiab] OR Osteoarthros*[tiab] OR Degenerative Arthritides[tiab] OR Degenerative Arthritis[tiab] OR Arthros*[tiab] OR “Inflammation Mediators”[MeSH Terms] OR “Inflammation”[MeSH Terms] OR inflammation [tiab] OR inflammaging[tiab] OR low grade inflammation[tiab] OR “Noncommunicable Diseases”[Mesh] OR “Chronic Disease”[Mesh] OR Chronic Disease*[tiab] OR Chronic Illness*[tiab] OR Chronically Ill[tiab] OR Noncommunicable Disease*[tiab] OR Non-infectious Disease*[tiab] OR Non infectious Disease*[tiab] OR Noninfectious Disease*[tiab] OR Non-communicable Chronic Disease*[tiab] OR “Coronary Disease”[Mesh] OR “Metabolic Syndrome”[Mesh] OR “Myocardial Infarction”[Mesh] OR “Cardiovascular Diseases”[Mesh] OR “Dyslipidemias”[Mesh] OR Coronary Disease*[tiab] OR Coronary Heart Disease*[tiab] OR Metabolic Syndrome*[tiab] OR Metabolic Cardiovascular Syndrome*[tiab] OR cardiovascular disease*[tiab] OR cvd[tiab] OR heart disease*[tiab] OR atherosclerosis[tiab] OR arteriosclerosis[tiab] OR cardiovascular event*[tiab] OR cardiovascular risk*[tiab] OR cardio-vascular event*[tiab] OR cardio-vascular risk*[tiab] OR dyslipidemia*[tiab] OR dys-lipidemia*[tiab] OR dyslipidaemia*[tiab] OR dys-lipidaemia*[tiab] OR “Overweight”[Mesh] OR obese*[tiab] OR overweight*[tiab] OR obesit*[tiab] OR “Diabetes, Gestational”[Mesh] OR “Diabetes Mellitus, Type 1”[Mesh] OR “Latent Autoimmune Diabetes in Adults”[Mesh] OR “Diabetes Mellitus”[Mesh] OR “Insulin Resistance”[Mesh] OR Insulin Sensitivity[tiab] OR Impaired Glucose Tolerance*[tiab] OR insulin resistan*[tiab] OR insuline resistan*[tiab] OR Hyperglycemia*[tiab] OR Hyper-glycemia*[tiab] OR Hyperglycaemia*[tiab] OR Hyper-glycaemia*[tiab] OR “Multiple Sclerosis”[Mesh] OR “Graves Ophthalmopathy”[Mesh] OR “Thyroiditis, Autoimmune”[Mesh] OR “Graves Disease”[Mesh] OR “Hashimoto Disease”[Mesh] OR “Colonic Neoplasms”[Mesh] OR “Sigmoid Neoplasms”[Mesh] OR “Colorectal Neoplasms”[Mesh] OR “Prostatic Neoplasms”[Mesh] OR “Breast Neoplasms”[Mesh] OR Colorectal Neoplasm*[tiab] OR Colorectal Carcinoma*[tiab] OR Colorectal Cancer*[tiab] OR Colorectal Tumor*[tiab] OR Prostate Neoplasm*[tiab] OR Prostatic Neoplasm*[tiab] OR Prostate Cancer*[tiab] OR Prostatic Cancer*[tiab] OR Breast Neoplasm*[tiab] OR Breast Tumor*[tiab] OR Breast Cancer*[tiab] OR Mammary Cancer*[tiab] OR Malignant Neoplasm of Breast*[tiab] OR Breast Carcinoma*[tiab] OR “Renal Insufficiency, Chronic”[Mesh] OR Chronic Renal	5,769,222

	Insufficienc*[tiab] OR Chronic Kidney Insufficienc*[tiab] OR Chronic Kidney Disease*[tiab] OR Chronic Renal Disease*[tiab]	
**#2**	“Microbiota”[Mesh:NoExp] OR “Gastrointestinal Microbiome”[Mesh] OR Microbiota*[tiab] OR Microbial Community[tiab] OR Microbial Communities[tiab] OR Microbiome*[tiab] OR Human Microbiome*[tiab] OR Gut Microbiome*[tiab] OR Gut Microflora*[tiab] OR Gut Microbiota*[tiab] OR Gastrointestinal Flora*[tiab] OR Gut Flora*[tiab] OR Gastrointestinal Microbiota*[tiab] OR Gastrointestinal Microflora*[tiab] OR Intestinal Microbiome*[tiab] OR Intestinal Microbiota*[tiab] OR Intestinal Microflora*[tiab] OR Intestinal Flora*[tiab]	106,777
**#1**	“Diet”[MeSH Terms] OR “Nutritional Sciences”[MeSH Terms] OR “Nutrition Therapy”[MeSH Terms] OR “Dietary Fiber”[Mesh] OR diet*[tiab] OR nutrition*[tiab] OR “Dietary Fiber”[tiab] OR “Wheat Bran”[tiab] OR Roughage*[tiab]	933,883

Embase.com Session Results 5 January 2021
/exp = EMtree keyword with explosion
/de = EMtree keyword without explosion
:ab,ti,kw = words in title or abstract or author keywords
/lim = Limit
Clinical trial: “Used for original reports of prospective clinical studies in which the (comparative) efficacy of one or more medical interventions in humans is evaluated”
Nonhuman: “Used for all items on non-human organisms (animals, bacteria, viruses, plants, etc.) or on tissue, cells or cell components from such organisms”
Normal human: “Used for original studies on normal (non-diseased) humans or normal human tissue”

#	**QUERY**	**RESULTS**
**#8**	#7 NOT ('animal'/exp NOT 'human'/exp)	1,610
**#7**	#6 AND ('clinical trial'/exp OR 'clinical trial (topic)'/exp OR randomi*:ab,ti,kw OR randomly*:ab,ti,kw OR trial:ab,ti,kw OR controls:ab,ti,kw OR ‘control group’:ab,ti,kw OR ‘controlled study’:ab,ti,kw)	2,097
**#6**	#5 AND [embase]/lim	9,436
**#5**	#4 NOT ([conference abstract]/lim OR [conference paper]/lim OR [conference review]/lim OR [editorial]/lim)	10,987
**#4**	#1 AND #2 AND #3	14,789
**#3**	‘inflammatory bowel disease’/exp OR ‘digestive system inflammation’/exp OR ‘inflammatory bowel disease’:ab,ti,kw OR ‘ulcerative colitis’:ab,ti,kw OR ‘crohns disease’:ab,ti,kw OR ‘Crohns Enteritis’:ab,ti,kw OR ‘Crohns Disease’:ab,ti,kw OR ‘Granulomatous Enteritis’:ab,ti,kw OR ‘Ileocolitis’:ab,ti,kw OR ‘Colitis, Granulomatous’:ab,ti,kw OR ‘Granulomatous Colitis’:ab,ti,kw OR ‘Terminal Ileitis’:ab,ti,kw OR ‘Regional Ileitis’:ab,ti,kw OR ‘Idiopathic Proctocolitis’:ab,ti,kw OR ‘Ulcerative Colitis’:ab,ti,kw OR ‘Colitis Gravis’:ab,ti,kw OR ‘autoimmune disease’/exp OR ‘Autoimmune disease*’:ab,ti,kw OR ‘Arthritides*’:ab,ti,kw OR ‘Polyarthritis’:ab,ti,kw OR ‘Polyarthritides’:ab,ti,kw OR ‘rheumatoid arthritis’:ab,ti,kw OR ‘Osteoarthritis’/exp OR Coxarthros*:ab,ti,kw OR Osteoarthritides:ab,ti,kw OR Osteoarthros*:ab,ti,kw OR ‘Degenerative Arthritides’:ab,ti,kw OR ‘Degenerative Arthritis’:ab,ti,kw OR Arthros*:ab,ti,kw OR ‘inflammation’/de OR ‘autoinflammatory disease’/de OR ‘inflammatory disease’/de OR inflammation:ab,ti,kw OR inflammaging:ab,ti,kw OR ‘low grade inflammation’:ab,ti,kw OR ‘chronic disease’/exp OR ‘non communicable disease’/de OR ‘Chronic Disease*’:ab,ti,kw OR ‘Chronic Illness*’:ab,ti,kw OR ‘Chronically Ill’:ab,ti,kw OR ‘Noncommunicable Disease’:ab,ti,kw OR ‘Non-infectious Disease*’:ab,ti,kw OR ‘Non infectious Disease*’:ab,ti,kw OR ‘Noninfectious Disease*’:ab,ti,kw OR ‘Non-communicable Chronic Disease*’:ab,ti,kw OR ‘coronary artery disease’/exp OR ‘ischemic heart disease’/exp OR ‘cardiovascular disease’/exp OR ‘dyslipidemia’/de OR ‘Coronary Disease*’:ab,ti,kw OR ‘Coronary Heart Disease*’:ab,ti,kw OR ‘Metabolic Syndrome*’:ab,ti,kw OR ‘Metabolic Cardiovascular Syndrome*’:ab,ti,kw OR ‘cardiovascular disease*’:ab,ti,kw OR cvd:ab,ti,kw OR ‘heart disease*’:ab,ti,kw OR ‘atherosclerosis’:ab,ti,kw OR ‘arteriosclerosis’:ab,ti,kw OR ‘cardiovascular event*’:ab,ti,kw OR ‘cardiovascular risk*’:ab,ti,kw OR ‘cardio-vascular event*’:ab,ti,kw OR ‘cardio-vascular risk*’:ab,ti,kw OR dyslipidemia*:ab,ti,kw OR ‘dys-lipidemia*’:ab,ti,kw OR	8,874,051

	dyslipidaemia*:ab,ti,kw OR dys-lipidaemia*:ab,ti,kw OR ‘obesity’/exp OR obese*:ab,ti,kw OR overweight*:ab,ti,kw OR obesit*:ab,ti,kw OR ‘diabetes mellitus’/exp OR ‘insulin resistance’/de OR ‘Insulin Sensitivity’:ab,ti,kw OR ‘Impaired Glucose Tolerance*’:ab,ti,kw OR ‘insulin resistan*’:ab,ti,kw OR ‘insuline resistan*’:ab,ti,kw OR ‘Hyperglycemia*’:ab,ti,kw OR ‘Hyper-glycemia*’:ab,ti,kw OR Hyperglycaemia*:ab,ti,kw OR ‘Hyper-glycaemia*’:ab,ti,kw OR ‘multiple sclerosis’/de OR ‘graves disease’/exp OR ‘autoimmune thyroiditis’/exp OR ‘prostate tumor’/exp OR ‘large intestine tumor’/exp OR ‘breast tumor’/exp OR ‘Colorectal Neoplasm*’:ab,ti,kw OR ‘Colorectal Carcinoma*’:ab,ti,kw OR ‘Colorectal Cancer*’:ab,ti,kw OR ‘Colorectal Tumor*’:ab,ti,kw OR ‘Prostate Neoplasm*’:ab,ti,kw OR ‘Prostate Cancer*’:ab,ti,kw OR ‘Prostatic Cancer*’:ab,ti,kw OR ‘Breast Neoplasm*’:ab,ti,kw OR ‘Breast Tumor*’:ab,ti,kw OR ‘Breast Cancer’:ab,ti,kw OR ‘Mammary Cancer*’:ab,ti,kw OR ‘Malignant Neoplasm of Breast’:ab,ti,kw OR ‘Breast Carcinoma*’:ab,ti,kw OR ‘chronic kidney failure’/exp OR ‘Chronic Renal Insufficienc*’:ab,ti,kw OR ‘Chronic Kidney Insufficienc*’:ab,ti,kw OR ‘Chronic Kidney Disease*’:ab,ti,kw OR ‘Chronic Renal Disease*’:ab,ti,kw	
**#2**	‘intestine flora’/de OR ‘microbiome’/exp OR ‘feces microflora’/de OR microbiota*:ab,ti,kw OR ‘Microbial Community’:ab,ti,kw OR ‘Microbial Communities’:ab,ti,kw OR Microbiome*:ab,ti,kw OR ‘Human Microbiome*’:ab,ti,kw OR ‘Gut Microbiome*’:ab,ti,kw OR ‘Gut Microflora’:ab,ti,kw OR ‘Gut Microbiota*’:ab,ti,kw OR ‘Gastrointestinal Flora’:ab,ti,kw OR ‘Gut Flora’:ab,ti,kw OR ‘Gastrointestinal Microbiota*’:ab,ti,kw OR ‘Gastrointestinal Microflora’:ab,ti,kw OR ‘Intestinal Microbiome*’:ab,ti,kw OR ‘Intestinal Microbiota*’:ab,ti,kw OR ‘Intestinal Microflora’:ab,ti,kw OR ‘Intestinal Flora’:ab,ti,kw	144,555
**#1**	'diet therapy'/exp OR 'diet'/exp OR diet*:ab,ti,kw OR nutrition*:ab,ti,kw OR ‘dietary Fiber*’:ab,ti,kw OR ‘wheat Bran*’:ab,ti,kw OR roughage*:ab,ti,kw	1,308,082

Wiley / Cochrane Library Session Results 5 January 2021

:ab,ti,kw = words in title or abstract or author keywords

#	QUERY	RESULTS
**#4**	#1 AND #2 AND #3 in Trials	1,445
**#3**	(“inflammatory bowel disease” OR “ulcerative colitis” OR “crohn’s disease” OR “Crohn's Enteritis” OR “Crohns Disease” OR “Granulomatous Enteritis” OR Ileocolitis OR “Colitis, Granulomatous” OR “Granulomatous Colitis” OR “Terminal Ileitis” OR “Regional Ileitis” OR “Idiopathic Proctocolitis” OR “Ulcerative Colitis” OR “Colitis Gravis” OR “Autoimmune Disease*” OR “systemic lupus erythematosus” OR SLE OR “Rheumatoid Arthritis” OR “arthritis, reactive” OR “reactive arthritis” OR “Myositis” OR “Sarcoidosis” OR “Behcet Syndrome” OR “spondylitis, ankylosing” OR “ankylosing spondylitis” OR “Psoriasis” OR Arthritides* OR Polyarthritis OR Polyarthritides OR “Osteoarthritis” OR Coxarthros* OR Osteoarthritides OR Osteoarthros* OR “Degenerative Arthritides” OR “Degenerative Arthritis” OR Arthros* OR “Inflammation Mediators” OR inflammation OR inflammaging OR “low grade inflammation” OR inflammatory OR Chronic Disease* OR Chronic Illness* OR “Chronically Ill” OR “Noncommunicable Disease*” OR “Non-infectious Disease*” OR “Non infectious Disease*” OR “Noninfectious Disease*” OR “Non-communicable Chronic Disease*” OR “Myocardial Infarction” OR “Coronary Disease*” OR “Coronary Heart Disease*” OR “Metabolic Syndrome*” OR “Metabolic Cardiovascular Syndrome*” OR “cardiovascular disease*” OR cvd OR “heart disease*” OR atherosclerosis OR arteriosclerosis OR “cardiovascular event*” OR “cardiovascular risk*” OR “cardio-vascular event*” OR “cardio-vascular risk*” OR dyslipidemia* OR dys-lipidemia* OR dyslipidaemia* OR dys-lipidaemia* OR obese* OR “overweight” OR “obesity” OR diabetes OR "diabetes mellitus" OR "insulin resistance" OR "impaired glucose tolerance" OR "insulin resistance" OR "hyperglycemia" OR "hyperglycaemia" OR “Multiple Sclerosis” OR “Graves Ophthalmopathy” OR “Thyroiditis, Autoimmune” OR “autoimmune thyroiditis” OR “Graves Disease” OR “Hashimoto Disease” OR “Colonic Neoplasms” OR “Sigmoid Neoplasms” OR “Colorectal Neoplasms” OR “Prostatic Neoplasms” OR “Breast Neoplasms” OR “Colorectal Neoplasm*” OR “Colorectal Carcinoma*” OR “Colorectal Cancer*” OR “Colorectal Tumor*” OR “Prostate Neoplasm*” OR “Prostatic Neoplasm*” OR “Prostate Cancer*” OR “Prostatic Cancer*” OR “Breast Neoplasm*” OR “Breast Tumor*” OR “Breast Cancer” OR “Mammary Cancer*” OR “Malignant Neoplasm” of Breast OR “Breast	429,414

	Carcinoma*” OR “Chronic Renal Insufficienc*” OR “Chronic Kidney Insufficienc*” OR “Chronic Kidney Disease*” OR “Chronic Renal Disease*”):ab,ti,kw	
**#2**	(Microbiota* OR “Microbial Community” OR “Microbial Communities” OR Microbiome* OR “Human Microbiome*” OR Gut Microbiome* OR “Gut Microflora” OR “Gut Microbiota*” OR “Gastrointestinal Flora” OR “Gut Flora” OR “Gastrointestinal Microbiota*” OR “Gastrointestinal Microflora” OR “Intestinal Microbiome*” OR “Intestinal Microbiota*” OR “Intestinal Microflora” OR “Intestinal Flora”):ab,ti,kw	7024
**#1**	(Diet* OR Nutrition* OR “Dietary Fiber*” OR “Wheat Bran*” OR Roughage*):ab,ti,kw	114,566

Ebsco / CINAHL Session Results 5 January 2021

MH = exact subject heading

+ = expload subject heading

TI = words in title

AB = words in abstract

#	**QUERY**	**RESULTS**
**S6**	S5 AND ((MH "Clinical Trials+") OR TI (“randomi*” OR “randomly*” OR “trial” OR “controls” OR “control group” OR “controlled study”) OR AB (“randomi*” OR “randomly*” OR “trial” OR “controls” OR “control group” OR “controlled study”))	711
**S5**	S4 NOT (MH “Animals” NOT MH “Human”)	3,274
**S4**	S1 AND S2 AND S3	3,552
**S3**	(MH arthritis) OR (MH “autoimmune diseases”) OR (MH osteoarthritis) OR (MH inflammation) OR (MH “chronic disease”) OR (MH “noncommunicable diseases”) OR (MH atherosclerosis) OR (MH obesity) OR (MH “diabetes mellitus”) OR (MH “insulin resistance”) OR (MH “hyperglycemia”) OR (MH multiple sclerosis) OR TI(“inflammatory bowel disease” OR “ulcerative colitis” OR “crohn’s disease” OR “Crohn's Enteritis” OR “Crohns Disease” OR “Granulomatous Enteritis” OR Ileocolitis OR “Colitis, Granulomatous” OR “Granulomatous Colitis” OR “Terminal Ileitis” OR “Regional Ileitis” OR “Idiopathic Proctocolitis” OR “Ulcerative Colitis” OR “Colitis Gravis” OR “Autoimmune Disease*” OR “systemic lupus erythematosus” OR SLE OR “Rheumatoid Arthritis” OR “arthritis, reactive” OR “reactive arthritis” OR “Myositis” OR “Sarcoidosis” OR “Behcet Syndrome” OR “spondylitis, ankylosing” OR “ankylosing spondylitis” OR “Psoriasis” OR Arthritides* OR Polyarthritis OR Polyarthritides OR “Osteoarthritis” OR Coxarthros* OR Osteoarthritides OR Osteoarthros* OR “Degenerative Arthritides” OR “Degenerative Arthritis” OR Arthros* OR “Inflammation Mediators” OR inflammation OR inflammaging OR “low grade inflammation” OR inflammatory OR Chronic Disease* OR Chronic Illness* OR “Chronically Ill” OR “Noncommunicable Disease*” OR “Non-infectious Disease*” OR “Non infectious Disease*” OR “Noninfectious Disease*” OR “Non-communicable Chronic Disease*” OR “Myocardial Infarction” OR “Coronary Disease*” OR “Coronary Heart Disease*” OR “Metabolic Syndrome*” OR “Metabolic Cardiovascular Syndrome*” OR “cardiovascular disease*” OR cvd OR “heart disease*” OR atherosclerosis OR arteriosclerosis OR “cardiovascular event*” OR “cardiovascular risk*” OR “cardio-vascular event*” OR “cardio-vascular risk*” OR dyslipidemia* OR dys-lipidemia* OR dyslipidaemia* OR dys-lipidaemia* OR “Diabetes, Gestational” OR “Diabetes” OR “Diabetes Mellitus” “OR Insulin Sensitivity” OR “Impaired Glucose Tolerance*” OR “insulin resistan*” OR “insuline resistan*” OR Hyperglycemia* OR Hyper-glycemia* OR Hyperglycaemia* OR Hyper-glycaemia* OR obese* OR overweight* OR obesit* OR “Multiple Sclerosis” OR “Graves Ophthalmopathy” OR “Thyroiditis, Autoimmune” OR “autoimmune thyroiditis” OR “Graves Disease” OR “Hashimoto Disease” OR “Colonic Neoplasms” OR “Sigmoid Neoplasms” OR “Colorectal Neoplasms” OR “Prostatic Neoplasms” OR “Breast Neoplasms” OR “Colorectal Neoplasm*” OR “Colorectal Carcinoma*” OR “Colorectal Cancer*” OR “Colorectal Tumor*” OR “Prostate Neoplasm*” OR “Prostatic Neoplasm*” OR “Prostate Cancer*” OR “Prostatic Cancer*” OR “Breast Neoplasm*” OR “Breast Tumor*” OR “Breast Cancer” OR “Mammary Cancer*” OR “Malignant Neoplasm” of Breast OR “Breast Carcinoma*” OR “Chronic Renal Insufficienc*” OR “Chronic Kidney Insufficienc*” OR “Chronic Kidney Disease*” OR “Chronic Renal Disease*”) OR AB(“inflammatory bowel disease” OR “ulcerative colitis” OR “crohn’s disease” OR “Crohn's Enteritis” OR “Crohns Disease” OR “Granulomatous	1,081,469

	Enteritis” OR Ileocolitis OR “Colitis, Granulomatous” OR “Granulomatous Colitis” OR “Terminal Ileitis” OR “Regional Ileitis” OR “Idiopathic Proctocolitis” OR “Ulcerative Colitis” OR “Colitis Gravis” OR “Autoimmune Disease*” OR “systemic lupus erythematosus” OR SLE OR “Rheumatoid Arthritis” OR “arthritis, reactive” OR “reactive arthritis” OR “Myositis” OR “Sarcoidosis” OR “Behcet Syndrome” OR “spondylitis, ankylosing” OR “ankylosing spondylitis” OR “Psoriasis” OR Arthritides* OR Polyarthritis OR Polyarthritides OR “Osteoarthritis” OR Coxarthros* OR Osteoarthritides OR Osteoarthros* OR “Degenerative Arthritides” OR “Degenerative Arthritis” OR Arthros* OR “Inflammation Mediators” OR inflammation OR inflammaging OR “low grade inflammation” OR inflammatory OR Chronic Disease* OR Chronic Illness* OR “Chronically Ill” OR “Noncommunicable Disease*” OR “Non-infectious Disease*” OR “Non infectious Disease*” OR “Noninfectious Disease*” OR “Non-communicable Chronic Disease*” OR “Myocardial Infarction” OR “Coronary Disease*” OR “Coronary Heart Disease*” OR “Metabolic Syndrome*” OR “Metabolic Cardiovascular Syndrome*” OR “cardiovascular disease*” OR cvd OR “heart disease*” OR atherosclerosis OR arteriosclerosis OR “cardiovascular event*” OR “cardiovascular risk*” OR “cardio-vascular event*” OR “cardio-vascular risk*” OR dyslipidemia* OR dys-lipidemia* OR dyslipidaemia* OR dys-lipidaemia* OR obese* OR overweight* OR obesit* OR “Diabetes, Gestational” OR “Diabetes” OR “Diabetes Mellitus” “OR Insulin Sensitivity” OR “Impaired Glucose Tolerance*” OR “insulin resistan*” OR “insuline resistan*” OR Hyperglycemia* OR Hyper-glycemia* OR Hyperglycaemia* OR Hyper-glycaemia* OR “Multiple Sclerosis” OR “Graves Ophthalmopathy” OR “Thyroiditis, Autoimmune” OR “autoimmune thyroiditis” OR “Graves Disease” OR “Hashimoto Disease” OR (“Colonic Neoplasms” OR “Sigmoid Neoplasms” OR “Colorectal Neoplasms” OR “Prostatic Neoplasms” OR “Breast Neoplasms” OR “Colorectal Neoplasm*” OR “Colorectal Carcinoma*” OR “Colorectal Cancer*” OR “Colorectal Tumor*” OR “Prostate Neoplasm*” OR “Prostatic Neoplasm*” OR “Prostate Cancer*” OR “Prostatic Cancer*” OR “Breast Neoplasm*” OR “Breast Tumor*” OR “Breast Cancer” OR “Mammary Cancer*” OR “Malignant Neoplasm” of Breast OR “Breast Carcinoma*” OR “Chronic Renal Insufficienc*” OR “Chronic Kidney Insufficienc*” OR “Chronic Kidney Disease*” OR “Chronic Renal Disease*”)	
**S2**	(MH microbiome or gut microbiota or intestinal or bacteria) OR TI (Microbiota* OR “Microbial Community” OR “Microbial Communities” OR Microbiome* OR “Human Microbiome*” OR Gut Microbiome* OR “Gut Microflora” OR “Gut Microbiota*” OR “Gastrointestinal Flora” OR “Gut Flora” OR “Gastrointestinal Microbiota*” OR “Gastrointestinal Microflora” OR “Intestinal Microbiome*” OR “Intestinal Microbiota*” OR “Intestinal Microflora” OR “Intestinal Flora”) OR AB (Microbiota* OR “Microbial Community” OR “Microbial Communities” OR Microbiome* OR “Human Microbiome*” OR Gut Microbiome* OR “Gut Microflora” OR “Gut Microbiota*” OR “Gastrointestinal Flora” OR “Gut Flora” OR “Gastrointestinal Microbiota*” OR “Gastrointestinal Microflora” OR “Intestinal Microbiome*” OR “Intestinal Microbiota*” OR “Intestinal Microflora” OR “Intestinal Flora”)	76,463
**S1**	(MH “Diet+”) OR (MH “Diet Therapy+”) OR (MH “Dietary Fiber”) OR TI (diet* OR dietet* OR “wheat bran*” OR roughage*) OR AB (diet* OR dietet* OR “wheat bran*” OR roughage*)	205,075

Total studies included: 4757
Duplicates removed: 1199
Studies after removal of duplicates: 3558

--

Second search including articles from 5 January 2021 to 2 July 2021
PubMed Session Results 2 July 2021

[Mesh terms] = Medical subject headings
[Mesh:NoExp] = Medical subject headings without explosion
[tiab] = words in title or abstract or author keywords

#	QUERY	RESULTS
**#7**	Date of publication in 2021	180
**#6**	#5 AND ("Clinical Trial" [Publication Type] OR "Clinical Trials as Topic"[Mesh] OR randomi*[tiab] OR randomly[tiab] OR trial[tiab] OR controls[tiab] OR “control group”[tiab] OR “controlled study”[tiab])	1,338
**#5**	#4 NOT (“Animals”[Mesh] NOT “Humans”[Mesh])	7,130
**#4**	#1 AND #2 AND #3	9,368
**#3**	“Autoimmune Diseases”[MeSH Terms] OR “Arthritis, Rheumatoid”[Mesh] OR “lupus erythematosus, systemic”[MeSH Terms] OR “arthritis, reactive”[MeSH Terms] OR “colitis, ulcerative”[MeSH Terms] OR “Inflammatory Bowel Diseases”[MeSH Terms] OR “Crohn Disease”[MeSH Terms] OR “Myositis”[MeSH Terms] OR “Sarcoidosis”[MeSH Terms] OR “Behcet Syndrome”[MeSH Terms] OR “spondylitis, ankylosing”[MeSH Terms] OR “Psoriasis”[Mesh] OR Autoimmune disease*[tiab] OR Arthritides*[tiab] OR Polyarthritis[tiab] OR Polyarthritides[tiab] OR inflammatory bowel disease*[tiab] OR ulcerative colitis[tiab] OR crohn’s disease*[tiab] OR Crohn's Enteritis[tiab] OR Crohns Disease*[tiab] OR Granulomatous Enteritis[tiab] OR Ileocolitis[tiab] OR Colitis, Granulomatous[tiab] OR Granulomatous Colitis[tiab] OR Terminal Ileitis[tiab] OR Regional Ileitis[tiab] OR Idiopathic Proctocolitis[tiab] OR Ulcerative Colitis[tiab] OR Colitis Gravis[tiab] OR “Osteoarthritis”[Mesh:NoExp] OR “Osteoarthritis, Hip”[Mesh] OR “Osteoarthritis, Knee”[Mesh] OR Coxarthros*[tiab] OR Osteoarthritides[tiab] OR Osteoarthros*[tiab] OR Degenerative Arthritides[tiab] OR Degenerative Arthritis[tiab] OR Arthros*[tiab] OR “Inflammation Mediators”[MeSH Terms] OR “Inflammation”[MeSH Terms] OR inflammation [tiab] OR inflammaging[tiab] OR low grade inflammation[tiab] OR “Noncommunicable Diseases”[Mesh] OR “Chronic Disease”[Mesh] OR Chronic Disease*[tiab] OR Chronic Illness*[tiab] OR Chronically Ill[tiab] OR Noncommunicable Disease*[tiab] OR Non-infectious Disease*[tiab] OR Non infectious Disease*[tiab] OR Noninfectious Disease*[tiab] OR Non-communicable Chronic Disease*[tiab] OR “Coronary Disease”[Mesh] OR “Metabolic Syndrome”[Mesh] OR “Myocardial Infarction”[Mesh] OR “Cardiovascular Diseases”[Mesh] OR “Dyslipidemias”[Mesh] OR Coronary Disease*[tiab] OR Coronary Heart Disease*[tiab] OR Metabolic Syndrome*[tiab] OR Metabolic Cardiovascular Syndrome*[tiab] OR cardiovascular disease*[tiab] OR cvd[tiab] OR heart disease*[tiab] OR atherosclerosis[tiab] OR arteriosclerosis[tiab] OR cardiovascular event*[tiab] OR cardiovascular risk*[tiab] OR cardio-vascular event*[tiab] OR cardio-vascular risk*[tiab] OR dyslipidemia*[tiab] OR dys-lipidemia*[tiab] OR dyslipidaemia*[tiab] OR dys-lipidaemia*[tiab] OR “Overweight”[Mesh] OR obese*[tiab] OR overweight*[tiab] OR obesit*[tiab] OR “Diabetes, Gestational”[Mesh] OR “Diabetes Mellitus, Type 1”[Mesh] OR “Latent Autoimmune Diabetes in Adults”[Mesh] OR “Diabetes Mellitus”[Mesh] OR “Insulin Resistance”[Mesh] OR Insulin Sensitivity[tiab] OR Impaired Glucose Tolerance*[tiab] OR insulin resistan*[tiab] OR insuline resistan*[tiab] OR Hyperglycemia*[tiab] OR Hyper-glycemia*[tiab] OR Hyperglycaemia*[tiab] OR Hyper-glycaemia*[tiab] OR “Multiple Sclerosis”[Mesh] OR “Graves Ophthalmopathy”[Mesh] OR “Thyroiditis, Autoimmune”[Mesh] OR “Graves Disease”[Mesh] OR “Hashimoto Disease”[Mesh] OR “Colonic Neoplasms”[Mesh] OR “Sigmoid Neoplasms”[Mesh] OR “Colorectal Neoplasms”[Mesh] OR “Prostatic Neoplasms”[Mesh] OR “Breast Neoplasms”[Mesh] OR Colorectal Neoplasm*[tiab] OR Colorectal Carcinoma*[tiab] OR Colorectal Cancer*[tiab] OR Colorectal Tumor*[tiab] OR Prostate Neoplasm*[tiab] OR Prostatic Neoplasm*[tiab] OR Prostate Cancer*[tiab] OR Prostatic Cancer*[tiab] OR Breast Neoplasm*[tiab] OR Breast Tumor*[tiab] OR Breast Cancer*[tiab] OR Mammary Cancer*[tiab] OR Malignant Neoplasm of Breast*[tiab] OR Breast Carcinoma*[tiab] OR “Renal Insufficiency, Chronic”[Mesh] OR Chronic Renal Insufficienc*[tiab] OR Chronic Kidney Insufficienc*[tiab] OR Chronic Kidney Disease*[tiab] OR Chronic Renal Disease*[tiab]	5,932,943
**#2**	“Microbiota”[Mesh:NoExp] OR “Gastrointestinal Microbiome”[Mesh] OR Microbiota*[tiab] OR Microbial Community[tiab] OR Microbial Communities[tiab] OR	118,234

	Microbiome*[tiab] OR Human Microbiome*[tiab] OR Gut Microbiome*[tiab] OR Gut Microflora*[tiab] OR Gut Microbiota*[tiab] OR Gastrointestinal Flora*[tiab] OR Gut Flora*[tiab] OR Gastrointestinal Microbiota*[tiab] OR Gastrointestinal Microflora*[tiab] OR Intestinal Microbiome*[tiab] OR Intestinal Microbiota*[tiab] OR Intestinal Microflora*[tiab] OR Intestinal Flora*[tiab]	
**#1**	"Diet"[MeSH Terms] OR "Nutritional Sciences"[MeSH Terms] OR "Nutrition Therapy"[MeSH Terms] OR "Dietary Fiber"[Mesh] OR diet*[tiab] OR nutrition*[tiab] OR "Dietary Fiber"[tiab] OR "Wheat Bran"[tiab] OR Roughage*[tiab]	963,744

Embase.com Session Results 2 July 2021
/exp = EMtree keyword with explosion
/de = EMtree keyword without explosion
:ab,ti,kw = words in title or abstract or author keywords
/lim = Limit
Clinical trial: "Used for original reports of prospective clinical studies in which the (comparative) efficacy of one or more medical interventions in humans is evaluated"
Nonhuman: "Used for all items on non-human organisms (animals, bacteria, viruses, plants, etc.) or on tissue, cells or cell components from such organisms"
Normal human: "Used for original studies on normal (non-diseased) humans or normal human tissue"

#	QUERY	RESULTS
**#9**	#7 NOT ('animal'/exp NOT 'human'/exp) AND [4-1-2021]/sd NOT [3-7-2021]/sd	237
**#8**	#7 NOT ('animal'/exp NOT 'human'/exp)	1,782
**#7**	#6 AND ('clinical trial'/exp OR 'clinical trial (topic)'/exp OR randomi*:ab,ti,kw OR randomly*:ab,ti,kw OR trial:ab,ti,kw OR controls:ab,ti,kw OR 'control group':ab,ti,kw OR 'controlled study':ab,ti,kw)	2,335
**#6**	#5 AND [embase]/lim	10,583
**#5**	#4 NOT ([conference abstract]/lim OR [conference paper]/lim OR [conference review]/lim OR [editorial]/lim)	12,362
**#4**	#1 AND #2 AND #3	16,422
**#3**	'inflammatory bowel disease'/exp OR 'digestive system inflammation'/exp OR 'inflammatory bowel disease':ab,ti,kw OR 'ulcerative colitis':ab,ti,kw OR 'crohns disease':ab,ti,kw OR 'Crohns Enteritis':ab,ti,kw OR 'Crohns Disease':ab,ti,kw OR 'Granulomatous Enteritis':ab,ti,kw OR 'Ileocolitis':ab,ti,kw OR 'Colitis, Granulomatous':ab,ti,kw OR 'Granulomatous Colitis':ab,ti,kw OR 'Terminal Ileitis':ab,ti,kw OR 'Regional Ileitis':ab,ti,kw OR 'Idiopathic Proctocolitis':ab,ti,kw OR 'Ulcerative Colitis':ab,ti,kw OR 'Colitis Gravis':ab,ti,kw OR 'autoimmune disease'/exp OR 'Autoimmune disease*':ab,ti,kw OR 'Arthritides*':ab,ti,kw OR 'Polyarthritis':ab,ti,kw OR 'Polyarthritides':ab,ti,kw OR 'rheumatoid arthritis':ab,ti,kw OR 'Osteoarthritis'/exp OR Coxarthros*:ab,ti,kw OR Osteoarthritides:ab,ti,kw OR Osteoarthros*:ab,ti,kw OR 'Degenerative Arthritides':ab,ti,kw OR 'Degenerative Arthritis':ab,ti,kw OR Arthros*:ab,ti,kw OR 'inflammation'/de OR 'autoinflammatory disease'/de OR 'inflammatory disease'/de OR inflammation:ab,ti,kw OR inflammaging:ab,ti,kw OR 'low grade inflammation':ab,ti,kw OR 'chronic disease'/exp OR 'non communicable disease'/de OR 'Chronic Disease*':ab,ti,kw OR 'Chronic Illness*':ab,ti,kw OR 'Chronically Ill':ab,ti,kw OR 'Noncommunicable Disease':ab,ti,kw OR 'Non-infectious Disease*':ab,ti,kw OR 'Non infectious Disease*':ab,ti,kw OR 'Noninfectious Disease*':ab,ti,kw OR 'Non-communicable Chronic Disease*':ab,ti,kw OR 'coronary artery disease'/exp OR 'ischemic heart disease'/exp OR 'cardiovascular disease'/exp OR 'dyslipidemia'/de OR 'Coronary Disease*':ab,ti,kw OR 'Coronary Heart Disease*':ab,ti,kw OR 'Metabolic Syndrome*':ab,ti,kw OR 'Metabolic Cardiovascular Syndrome*':ab,ti,kw OR 'cardiovascular disease*':ab,ti,kw OR cvd:ab,ti,kw OR 'heart disease*':ab,ti,kw OR 'atherosclerosis':ab,ti,kw OR 'arteriosclerosis':ab,ti,kw OR 'cardiovascular event*':ab,ti,kw OR 'cardiovascular risk*':ab,ti,kw OR 'cardio-vascular event*':ab,ti,kw OR 'cardio-vascular risk*':ab,ti,kw OR dyslipidemia*:ab,ti,kw OR 'dys-lipidemia*':ab,ti,kw OR dyslipidaemia*:ab,ti,kw OR dys-lipidaemia*:ab,ti,kw OR 'obesity'/exp OR obese*:ab,ti,kw OR overweight*:ab,ti,kw OR obesit*:ab,ti,kw OR 'diabetes mellitus'/exp OR 'insulin resistance'/de OR 'Insulin Sensitivity':ab,ti,kw OR 'Impaired Glucose Tolerance*':ab,ti,kw	9,145,638

	OR 'insulin resistan*':ab,ti,kw OR 'insuline resistan*':ab,ti,kw OR 'Hyperglycemia*':ab,ti,kw OR 'Hyper-glycemia*':ab,ti,kw OR Hyperglycaemia*:ab,ti,kw OR 'Hyper-glycaemia*':ab,ti,kw OR 'multiple sclerosis'/de OR 'graves disease'/exp OR 'autoimmune thyroiditis'/exp OR 'prostate tumor'/exp OR 'large intestine tumor'/exp OR 'breast tumor'/exp OR 'Colorectal Neoplasm*':ab,ti,kw OR 'Colorectal Carcinoma*':ab,ti,kw OR 'Colorectal Cancer*':ab,ti,kw OR 'Colorectal Tumor*':ab,ti,kw OR 'Prostate Neoplasm*':ab,ti,kw OR 'Prostate Cancer*':ab,ti,kw OR 'Prostatic Cancer*':ab,ti,kw OR 'Breast Neoplasm*':ab,ti,kw OR 'Breast Tumor*':ab,ti,kw OR 'Breast Cancer':ab,ti,kw OR 'Mammary Cancer*':ab,ti,kw OR 'Malignant Neoplasm of Breast':ab,ti,kw OR 'Breast Carcinoma*':ab,ti,kw OR 'chronic kidney failure'/exp OR 'Chronic Renal Insufficienc*':ab,ti,kw OR 'Chronic Kidney Insufficienc*':ab,ti,kw OR 'Chronic Kidney Disease*':ab,ti,kw OR 'Chronic Renal Disease*':ab,ti,kw	
#2	'intestine flora'/de OR 'microbiome'/exp OR 'feces microflora'/de OR microbiota*:ab,ti,kw OR 'Microbial Community':ab,ti,kw OR 'Microbial Communities':ab,ti,kw OR Microbiome*:ab,ti,kw OR 'Human Microbiome*':ab,ti,kw OR 'Gut Microbiome*':ab,ti,kw OR 'Gut Microflora':ab,ti,kw OR 'Gut Microbiota*':ab,ti,kw OR 'Gastrointestinal Flora':ab,ti,kw OR 'Gut Flora':ab,ti,kw OR 'Gastrointestinal Microbiota*':ab,ti,kw OR 'Gastrointestinal Microflora':ab,ti,kw OR 'Intestinal Microbiome*':ab,ti,kw OR 'Intestinal Microbiota*':ab,ti,kw OR 'Intestinal Microflora':ab,ti,kw OR 'Intestinal Flora':ab,ti,kw	158,178
#1	'diet therapy'/exp OR 'diet'/exp OR diet*:ab,ti,kw OR nutrition*:ab,ti,kw OR 'dietary Fiber*':ab,ti,kw OR 'wheat Bran*':ab,ti,kw OR roughage*:ab,ti,kw	1,350,657

Wiley / Cochrane Library Session Results 2 July 2021

:ab,ti,kw = words in title or abstract or author keywords

#	**QUERY**	**RESULTS**
#5	Custom data rage 04-01-2021 – 02-07-2021	175
#4	#1 AND #2 AND #3 in Trials	1,626
#3	("inflammatory bowel disease" OR "ulcerative colitis" OR "crohn's disease" OR "Crohn's Enteritis" OR "Crohns Disease" OR "Granulomatous Enteritis" OR Ileocolitis OR "Colitis, Granulomatous" OR "Granulomatous Colitis" OR "Terminal Ileitis" OR "Regional Ileitis" OR "Idiopathic Proctocolitis" OR "Ulcerative Colitis" OR "Colitis Gravis" OR "Autoimmune Disease*" OR "systemic lupus erythematosus" OR SLE OR "Rheumatoid Arthritis" OR "arthritis, reactive" OR "reactive arthritis" OR "Myositis" OR "Sarcoidosis" OR "Behcet Syndrome" OR "spondylitis, ankylosing" OR "ankylosing spondylitis" OR "Psoriasis" OR Arthritides* OR Polyarthritis OR Polyarthritides OR "Osteoarthritis" OR Coxarthros* OR Osteoarthritides OR Osteoarthros* OR "Degenerative Arthritides" OR "Degenerative Arthritis" OR Arthros* OR "Inflammation Mediators" OR inflammation OR inflammaging OR "low grade inflammation" OR inflammatory OR Chronic Disease* OR Chronic Illness* OR "Chronically Ill" OR "Noncommunicable Disease*" OR "Non-infectious Disease*" OR "Non infectious Disease*" OR "Noninfectious Disease*" OR "Non-communicable Chronic Disease*" OR "Myocardial Infarction" OR "Coronary Disease*" OR "Coronary Heart Disease*" OR "Metabolic Syndrome*" OR "Metabolic Cardiovascular Syndrome*" OR "cardiovascular disease*" OR cvd OR "heart disease*" OR atherosclerosis OR arteriosclerosis OR "cardiovascular event*" OR "cardiovascular risk*" OR "cardio-vascular event*" OR "cardio-vascular risk*" OR dyslipidemia* OR dys-lipidemia* OR dyslipidaemia* OR dys-lipidaemia* OR obese* OR "overweight" OR "obesity" OR diabetes OR "diabetes mellitus" OR "insulin resistance" OR "impaired glucose tolerance" OR "insulin resistance" OR "hyperglycemia" OR "hyperglycaemia" OR "Multiple Sclerosis" OR "Graves Ophthalmopathy" OR "Thyroiditis, Autoimmune" OR "autoimmune thyroiditis" OR "Graves Disease" OR "Hashimoto Disease" OR "Colonic Neoplasms" OR "Sigmoid Neoplasms" OR "Colorectal Neoplasms" OR "Prostatic Neoplasms" OR "Breast Neoplasms" OR "Colorectal Neoplasm*" OR "Colorectal Carcinoma*" OR "Colorectal Cancer*" OR "Colorectal Tumor*" OR "Prostate Neoplasm*" OR "Prostatic Neoplasm*" OR "Prostate Cancer*" OR "Prostatic Cancer*" OR "Breast Neoplasm*" OR "Breast Tumor*" OR "Breast Cancer" OR "Mammary Cancer*" OR "Malignant Neoplasm" of Breast OR "Breast Carcinoma*" OR "Chronic Renal Insufficienc*" OR "Chronic Kidney Insufficienc*" OR "Chronic Kidney Disease*" OR "Chronic Renal Disease*"):ab,ti,kw	447,259

**#2**	(Microbiota* OR "Microbial Community" OR "Microbial Communities" OR Microbiome* OR "Human Microbiome*" OR Gut Microbiome* OR "Gut Microflora" OR "Gut Microbiota*" OR "Gastrointestinal Flora" OR "Gut Flora" OR "Gastrointestinal Microbiota*" OR "Gastrointestinal Microflora" OR "Intestinal Microbiome*" OR "Intestinal Microbiota*" OR "Intestinal Microflora" OR "Intestinal Flora"):ab,ti,kw	7,724
**#1**	(Diet* OR Nutrition* OR "Dietary Fiber*" OR "Wheat Bran*" OR Roughage*):ab,ti,kw	119,159

Ebsco / CINAHL Session Results 2 July 2021
MH = exact subject heading
+ = expload subject heading
TI = words in title
AB = words in abstract

#	**QUERY**	**RESULTS**
**S7**	S6 Limiters - Published Date: 20210101-20210731	65
**S6**	S5 AND ((MH "Clinical Trials+") OR TI ("randomi*" OR "randomly*" OR "trial" OR "controls" OR "control group" OR "controlled study") OR AB ("randomi*" OR "randomly*" OR "trial" OR "controls" OR "control group" OR "controlled study"))	779
**S5**	S4 NOT (MH "Animals" NOT MH "Human")	3,595
**S4**	S1 AND S2 AND S3	3,884
**S3**	(MH arthritis) OR (MH "autoimmune diseases") OR (MH osteoarthritis) OR (MH inflammation) OR (MH "chronic disease") OR (MH "noncommunicable diseases") OR (MH atherosclerosis) OR (MH obesity) OR (MH "diabetes mellitus") OR (MH "insulin resistance") OR (MH "hyperglycemia") OR (MH multiple sclerosis) OR TI("inflammatory bowel disease" OR "ulcerative colitis" OR "crohn's disease" OR "Crohn's Enteritis" OR "Crohns Disease" OR "Granulomatous Enteritis" OR Ileocolitis OR "Colitis, Granulomatous" OR "Granulomatous Colitis" OR "Terminal Ileitis" OR "Regional Ileitis" OR "Idiopathic Proctocolitis" OR "Ulcerative Colitis" OR "Colitis Gravis" OR "Autoimmune Disease*" OR "systemic lupus erythematosus" OR SLE OR "Rheumatoid Arthritis" OR "arthritis, reactive" OR "reactive arthritis" OR "Myositis" OR "Sarcoidosis" OR "Behcet Syndrome" OR "spondylitis, ankylosing" OR "ankylosing spondylitis" OR "Psoriasis" OR Arthritides* OR Polyarthritis OR Polyarthritides OR "Osteoarthritis" OR Coxarthros* OR Osteoarthritides OR Osteoarthros* OR "Degenerative Arthritides" OR "Degenerative Arthritis" OR Arthros* OR "Inflammation Mediators" OR inflammation OR inflammaging OR "low grade inflammation" OR inflammatory OR Chronic Disease* OR Chronic Illness* OR "Chronically Ill" OR "Noncommunicable Disease*" OR "Non-infectious Disease*" OR "Non infectious Disease*" OR "Noninfectious Disease*" OR "Non-communicable Chronic Disease*" OR "Myocardial Infarction" OR "Coronary Disease*" OR "Coronary Heart Disease*" OR "Metabolic Syndrome*" OR "Metabolic Cardiovascular Syndrome*" OR "cardiovascular disease*" OR cvd OR "heart disease*" OR atherosclerosis OR arteriosclerosis OR "cardiovascular event*" OR "cardiovascular risk*" OR "cardio-vascular event*" OR "cardio-vascular risk*" OR dyslipidemia* OR dys-lipidemia* OR dyslipidaemia* OR dys-lipidaemia* OR "Diabetes, Gestational" OR "Diabetes" OR "Diabetes Mellitus" "OR Insulin Sensitivity" OR "Impaired Glucose Tolerance*" OR "insulin resistan*" OR "insuline resistan*" OR Hyperglycemia* OR Hyper-glycemia* OR Hyperglycaemia* OR Hyper-glycaemia* OR obese* OR overweight* OR obesit* OR "Multiple Sclerosis" OR "Graves Ophthalmopathy" OR "Thyroiditis, Autoimmune" OR "autoimmune thyroiditis" OR "Graves Disease" OR "Hashimoto Disease" OR "Colonic Neoplasms" OR "Sigmoid Neoplasms" OR "Colorectal Neoplasms" OR "Prostatic Neoplasms" OR "Breast Neoplasms" OR "Colorectal Neoplasm*" OR "Colorectal Carcinoma*" OR "Colorectal Cancer*" OR "Colorectal Tumor*" OR "Prostate Neoplasm*" OR "Prostatic Neoplasm*" OR "Prostate Cancer*" OR "Prostatic Cancer*" OR "Breast Neoplasm*" OR "Breast Tumor*" OR "Breast Cancer" OR "Mammary Cancer*" OR "Malignant Neoplasm" of Breast OR "Breast Carcinoma*" OR "Chronic Renal Insufficienc*" OR "Chronic Kidney Insufficienc*" OR "Chronic Kidney Disease*" OR "Chronic Renal Disease*") OR AB("inflammatory bowel disease" OR "ulcerative colitis" OR "crohn's disease" OR "Crohn's Enteritis" OR "Crohns Disease" OR "Granulomatous Enteritis" OR Ileocolitis OR "Colitis, Granulomatous" OR "Granulomatous Colitis" OR	1,119,212

	"Terminal Ileitis" OR "Regional Ileitis" OR "Idiopathic Proctocolitis" OR "Ulcerative Colitis" OR "Colitis Gravis" OR "Autoimmune Disease*" OR "systemic lupus erythematosus" OR SLE OR "Rheumatoid Arthritis" OR "arthritis, reactive" OR "reactive arthritis" OR "Myositis" OR "Sarcoidosis" OR "Behcet Syndrome" OR "spondylitis, ankylosing" OR "ankylosing spondylitis" OR "Psoriasis" OR Arthritides* OR Polyarthritis OR Polyarthritides OR "Osteoarthritis" OR Coxarthros* OR Osteoarthritides OR Osteoarthros* OR "Degenerative Arthritides" OR "Degenerative Arthritis" OR Arthros* OR "Inflammation Mediators" OR inflammation OR inflammaging OR "low grade inflammation" OR inflammatory OR Chronic Disease* OR Chronic Illness* OR "Chronically Ill" OR "Noncommunicable Disease*" OR "Non-infectious Disease*" OR "Non infectious Disease*" OR "Noninfectious Disease*" OR "Non-communicable Chronic Disease*" OR "Myocardial Infarction" OR "Coronary Disease*" OR "Coronary Heart Disease*" OR "Metabolic Syndrome*" OR "Metabolic Cardiovascular Syndrome*" OR "cardiovascular disease*" OR cvd OR "heart disease*" OR atherosclerosis OR arteriosclerosis OR "cardiovascular event*" OR "cardiovascular risk*" OR "cardio-vascular event*" OR "cardio-vascular risk*" OR dyslipidemia* OR dys-lipidemia* OR dyslipidaemia* OR dys-lipidaemia* OR obese* OR overweight* OR obesit* OR "Diabetes, Gestational" OR "Diabetes" OR "Diabetes Mellitus" "OR Insulin Sensitivity" OR "Impaired Glucose Tolerance*" OR "insulin resistan*" OR "insuline resistan*" OR Hyperglycemia* OR Hyper-glycemia* OR Hyperglycaemia* OR Hyper-glycaemia* OR "Multiple Sclerosis" OR "Graves Ophthalmopathy" OR "Thyroiditis, Autoimmune" OR "autoimmune thyroiditis" OR "Graves Disease" OR "Hashimoto Disease" OR ("Colonic Neoplasms" OR "Sigmoid Neoplasms" OR "Colorectal Neoplasms" OR "Prostatic Neoplasms" OR "Breast Neoplasms" OR "Colorectal Neoplasm*" OR "Colorectal Carcinoma*" OR "Colorectal Cancer*" OR "Colorectal Tumor*" OR "Prostate Neoplasm*" OR "Prostatic Neoplasm*" OR "Prostate Cancer*" OR "Prostatic Cancer*" OR "Breast Neoplasm*" OR "Breast Tumor*" OR "Breast Cancer" OR "Mammary Cancer*" OR "Malignant Neoplasm" of Breast OR "Breast Carcinoma*" OR "Chronic Renal Insufficienc*" OR "Chronic Kidney Insufficienc*" OR "Chronic Kidney Disease*" OR "Chronic Renal Disease*")	
**S2**	(MH microbiome or gut microbiota or intestinal or bacteria) OR TI (Microbiota* OR "Microbial Community" OR "Microbial Communities" OR Microbiome* OR "Human Microbiome*" OR Gut Microbiome* OR "Gut Microflora" OR "Gut Microbiota*" OR "Gastrointestinal Flora" OR "Gut Flora" OR "Gastrointestinal Microbiota*" OR "Gastrointestinal Microflora" OR "Intestinal Microbiome*" OR "Intestinal Microbiota*" OR "Intestinal Microflora" OR "Intestinal Flora") OR AB (Microbiota* OR "Microbial Community" OR "Microbial Communities" OR Microbiome* OR "Human Microbiome*" OR Gut Microbiome* OR "Gut Microflora" OR "Gut Microbiota*" OR "Gastrointestinal Flora" OR "Gut Flora" OR "Gastrointestinal Microbiota*" OR "Gastrointestinal Microflora" OR "Intestinal Microbiome*" OR "Intestinal Microbiota*" OR "Intestinal Microflora" OR "Intestinal Flora")	79,825
**S1**	(MH "Diet+") OR (MH "Diet Therapy+") OR (MH "Dietary Fiber") OR TI (diet* OR dietet* OR "wheat bran*" OR roughage*) OR AB (diet* OR dietet* OR "wheat bran*" OR roughage*)	212,090

Total studies included: 657
Duplicates removed: 241
Studies after removal of duplicates:

- **Pubmed: 173**
- **Embase: 105**
- **Cochrane: 112**
- **CINAHL: 26**

Supplementary Figures S1-3: Risk of Bias assessment

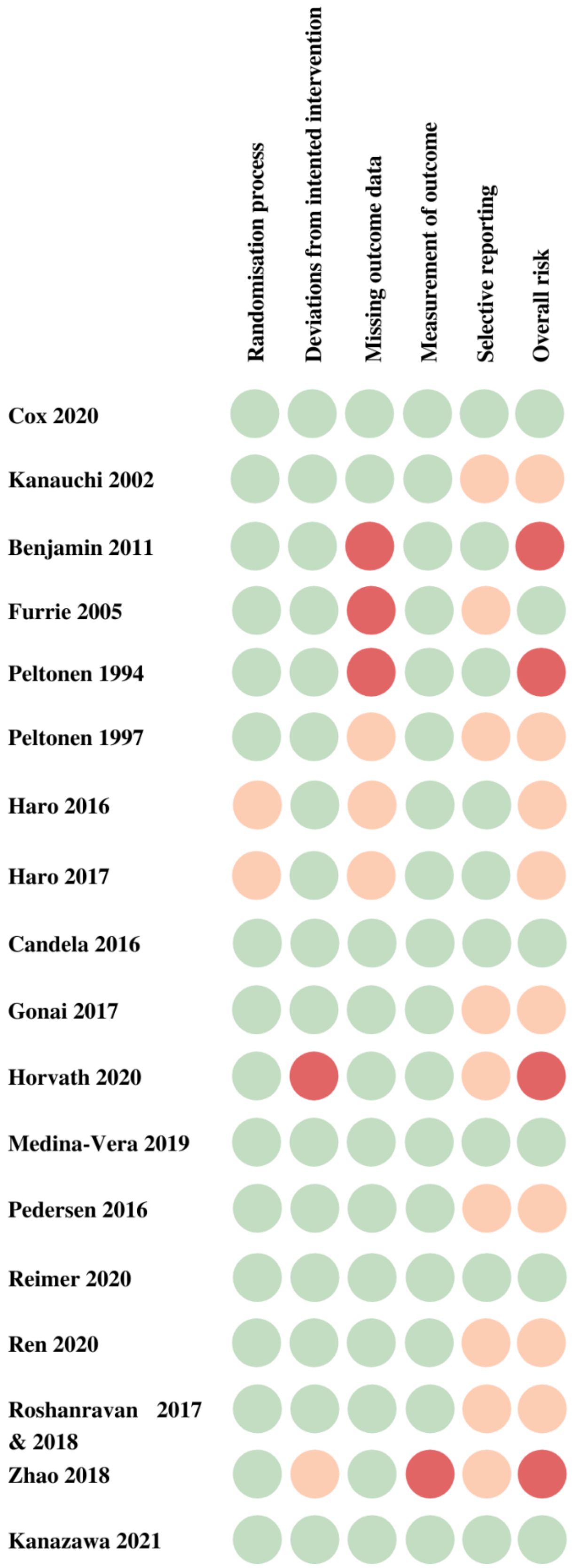

Figure S1. Risk of bias assessment of randomized clinical trials included in this review according to the Revised Cochrane risk-of-bias tool for randomized trials (RoB 2). Red: high risk, orange: moderate risk, green: low risk.

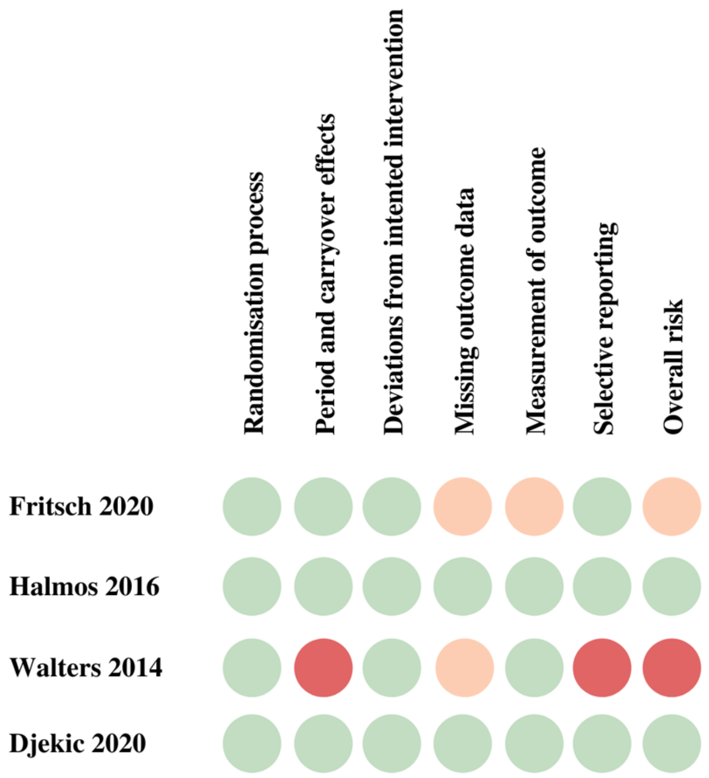

Figure S2. Risk of bias assessment of randomized crossover trials included in this review according to the Revised Cochrane risk-of-bias tool for randomized crossover trials. Red: high risk, orange: moderate risk, green: low risk.

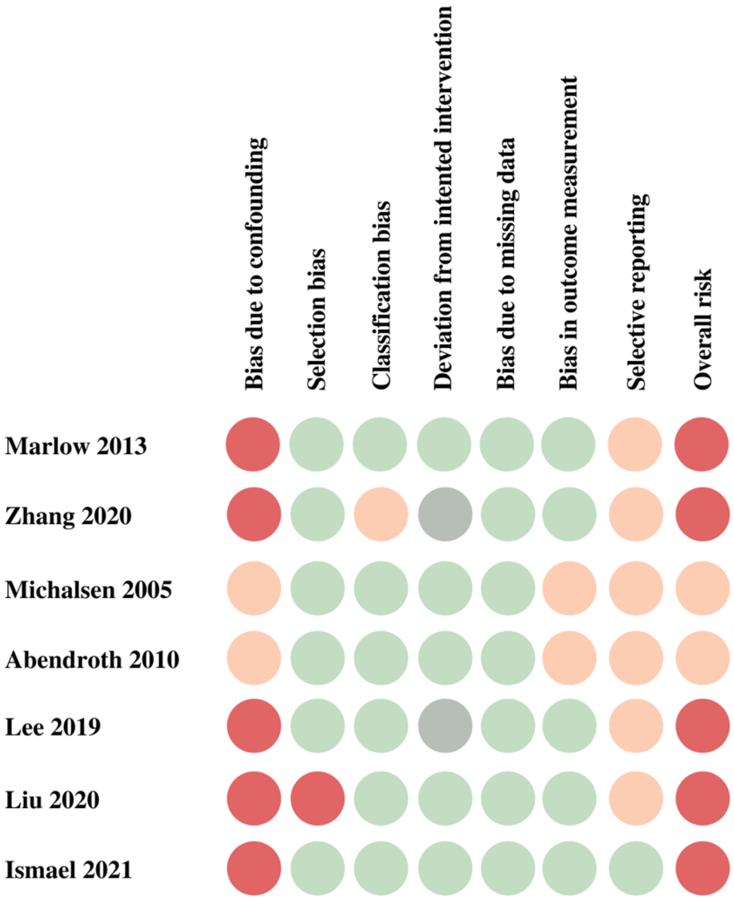

Figure S3. Risk of bias assessment of non-randomized trials included in this review according to the Risk of Bias in Non-randomized Studies-of Interventions (ROBINS-I) assessment tool. Red: high/critical risk, orange: moderate risk, green: low risk, grey: no information or unclear risk.
